# Physiological and pathological roles of *FATP*-mediated lipid droplets in *Drosophila* and mice retina

**DOI:** 10.1371/journal.pgen.1007627

**Published:** 2018-09-10

**Authors:** Daan M. Van Den Brink, Aurélie Cubizolle, Gilles Chatelain, Nathalie Davoust, Victor Girard, Simone Johansen, Francesco Napoletano, Pierre Dourlen, Laurent Guillou, Claire Angebault-Prouteau, Nathalie Bernoud-Hubac, Michel Guichardant, Philippe Brabet, Bertrand Mollereau

**Affiliations:** 1 Université de Lyon, ENSL, UCBL, CNRS, LBMC, UMS 3444 Biosciences Lyon Gerland, Lyon, France; 2 Institut des Neurosciences de Montpellier, INSERM U1051, CHU St Eloi, Montpellier, France; 3 Université de Montpellier, Montpellier, France; 4 Molecular Oncology Unit, Department of Life Sciences, University of Trieste c/o Laboratorio Nazionale CIB, Area Science Park, Trieste, Italy; 5 Institut Pasteur de Lille; Inserm, U1167, RID-AGE-Risk Factors and Molecular Determinants of Aging-Related Diseases; University Lille, U1167-Excellence Laboratory LabEx DISTALZ, Lille, France; 6 INSERM U1046, UMR CNRS 9214, Université de Montpellier, CHRU de Montpellier, Montpellier, France; 7 Univ Lyon, CarMeN laboratory, INSA Lyon, INSERM U1060, INRA U1397, Université Claude Bernard Lyon 1, F-69621, Villeurbanne, France; Indiana University, UNITED STATES

## Abstract

Increasing evidence suggests that dysregulation of lipid metabolism is associated with neurodegeneration in retinal diseases such as age-related macular degeneration and in brain disorders such as Alzheimer’s and Parkinson’s diseases. Lipid storage organelles (lipid droplets, LDs), accumulate in many cell types in response to stress, and it is now clear that LDs function not only as lipid stores but also as dynamic regulators of the stress response. However, whether these LDs are always protective or can also be deleterious to the cell is unknown. Here, we investigated the consequences of LD accumulation on retinal cell homeostasis under physiological and stress conditions in *Drosophila* and in mice. In wild-type *Drosophila*, we show that *dFatp* is required and sufficient for expansion of LD size in retinal pigment cells (RPCs) and that LDs in RPCs are required for photoreceptor survival during aging. Similarly, in mice, LD accumulation induced by RPC-specific expression of human *FATP1* was non-toxic and promoted mitochondrial energy metabolism in RPCs and non-autonomously in photoreceptor cells. In contrast, the inhibition of LD accumulation by *dFatp* knockdown suppressed neurodegeneration in *Aats-met*^*FB*^ *Drosophila* mutants, which carry elevated levels of reactive oxygen species (ROS). This suggests that abnormal turnover of LD may be toxic for photoreceptors cells of the retina under oxidative stress. Collectively, these findings indicate that *FATP*-mediated LD formation in RPCs promotes RPC and neuronal homeostasis under physiological conditions but could be deleterious for the photoreceptors under pathological conditions.

## Author summary

Lipids are major cell constituents and are present in the membranes, as free lipids in the cytoplasm, or stored in vesicles called lipid droplets (LDs). Under conditions of stress, lipids stored in LDs can be released to serve as substrates for energy metabolism by mitochondria. However, lipid storage is dysregulated in many degenerative disorders such as age-related macular degeneration, Parkinson’s and Alzheimer’s diseases. Thus, it is unclear whether accumulation of LDs is protective or toxic for neuronal cells. To address this question, we examined the consequences of removal or enforced LD accumulation on the health of retinal cells in flies and mice. Like humans, fly and mouse retinas contain retinal pigment cells (RPC) that support the functions of neighboring photoreceptor cells. We found that overexpression of the fatty acid transport protein (FATP) in RPCs induced accumulation of LDs in both transgenic flies and mice. Moreover, LD accumulation in RPCs was not harmful for juxtaposed photoreceptors during aging, but was toxic under stress conditions. We propose that lipid storage promotes cellular communication that affects photoreceptor health.

## Introduction

Photoreceptor neurons are among the highest energy consumers in the body. They are sustained by a layer of retinal pigment epithelial cells (here termed retinal pigment cells [RPC], S1 Fig) that provide photoreceptors with a constant supply of substrates for energy production by mitochondrial oxidation via the tricarboxylic acid (TCA) cycle. An inability of RPCs to perform this role leads to photoreceptor death and is associated with diseases such as age-related macular degeneration (AMD), the most prevalent cause of blindness in developed countries [1].

Injury to RPCs, whether through disease or as part of the normal aging process, is accompanied by an accumulation of lipid deposits, named drusen, in the RPC itself or in the adjacent Bruch’s membrane [2–4]. A common cause of injury is reduced blood flow to the retina, which causes hypoxia and a subsequent metabolic shift that results in accumulation in RPCs of intracellular lipid droplets (LDs) composed of neutral lipids, such as triacylglycerides (TAGs) and sterol esters. In AMD, lipids are also deposited extracellularly, further compromising the functions of RPCs and photoreceptors [5]. However, it is not clear whether lipid accumulation is a cause or a consequence of AMD pathology.

Work in *Drosophila* models has contributed greatly to our understanding of the mechanisms of LD biosynthesis and physiological functions [6, 7]. For example, LDs have been shown to play an antioxidant role in the developing *Drosophila* nervous system by protecting polyunsaturated fatty acids from peroxidation under conditions of hypoxia [8]. In contrast, in *Drosophila* carrying mutations with mitochondrial dysfunction, oxidative stress induces glial cells to accumulate LDs that are toxic to the neighboring photoreceptor cells [9]. Thus, LDs are not simply cytoplasmic lipid storage organelles with critical roles in energy metabolism; they also have dynamic functions in regulating the response to stress of many cell types, including those in the nervous system [10–12].

LDs are synthesized on the endoplasmic reticulum membrane by a protein complex composed of diacylglycerol acyltransferase (DGAT-2) and fatty acid transport protein (FATP) [12, 13]. The FATP family (also known as solute carrier family 27 [SLC27]) [14, 15] are acyl-CoA synthetases involved in the cellular import of FAs [16] as well as other processes. FATPs interact physically and functionally with DGAT-2, an enzyme that catalyzes the conjugation of a fatty acyl-CoA to diacyl glycerol to produce TAGs, which are then incorporated into expanding LDs in *C*. *eleg*ans [13]. An important role for *FATP* in TAG storage has also been demonstrated in mammals. In *Fatp1* knockout mice, the uptake of FA and the size of LDs in the brown adipose tissue are reduced compared with wild-type (WT) mice, resulting in defects in non-shivering thermogenesis [17]. The *FATP1* gene is conserved from yeast to humans, where there are six proteins with different tissue distributions and substrate preferences. *Drosophila Fatp* (*dFatp*) shows a high degree of sequence similarity to human (*h*)*FATP1* and *hFATP4*, which have broad expression profiles that include the brain and retina [16, 18, 19].

The *Drosophila* retina is composed of approximately 800 ommatidia, each of which comprises eight photoreceptors surrounded by a hexagonal lattice of nine RPCs (six secondary and three tertiary pigment cells, S1 Fig) [20]. *Drosophila* RPCs (dRPCs) differentiate during the pupal stage and superfluous cells are eliminated by apoptosis [21, 22]. The survival of dRPCs requires cues that are provided by cell-to-cell communication from neighboring cone and primary pigment cells during pupal development [23, 24]. In adult flies, dRPCs have similar functions to mammalian RPCs; that is, they are also juxtaposed to photoreceptor cells and supply metabolites for energy metabolism and other functions [25, 26, 27].

In *Drosophila*, *dFatp* is expressed in both photoreceptors and dRPCs [28], but its physiological role in LD formation in dRPCs and its impact on photoreceptor homeostasis remains to be investigated. Here, we examined the role of *FATP* in LDs formation and in cellular retinal homeostasis in *Drosophila* and in mice. We find that, although the architecture of the mammalian and insect retina are different, RPCs in both organisms play conserved roles in lipid and photoreceptor homeostasis. We show that *FATP* plays a crucial role in regulating LD production in RPCs and, in turn, RPC-derived LDs affect differentially photoreceptor viability in physiological and pathological conditions.

## Results

### *dFatp* is necessary and sufficient for LD expansion in *Drosophila* retina under physiological and pathological conditions

**Fig 1:**
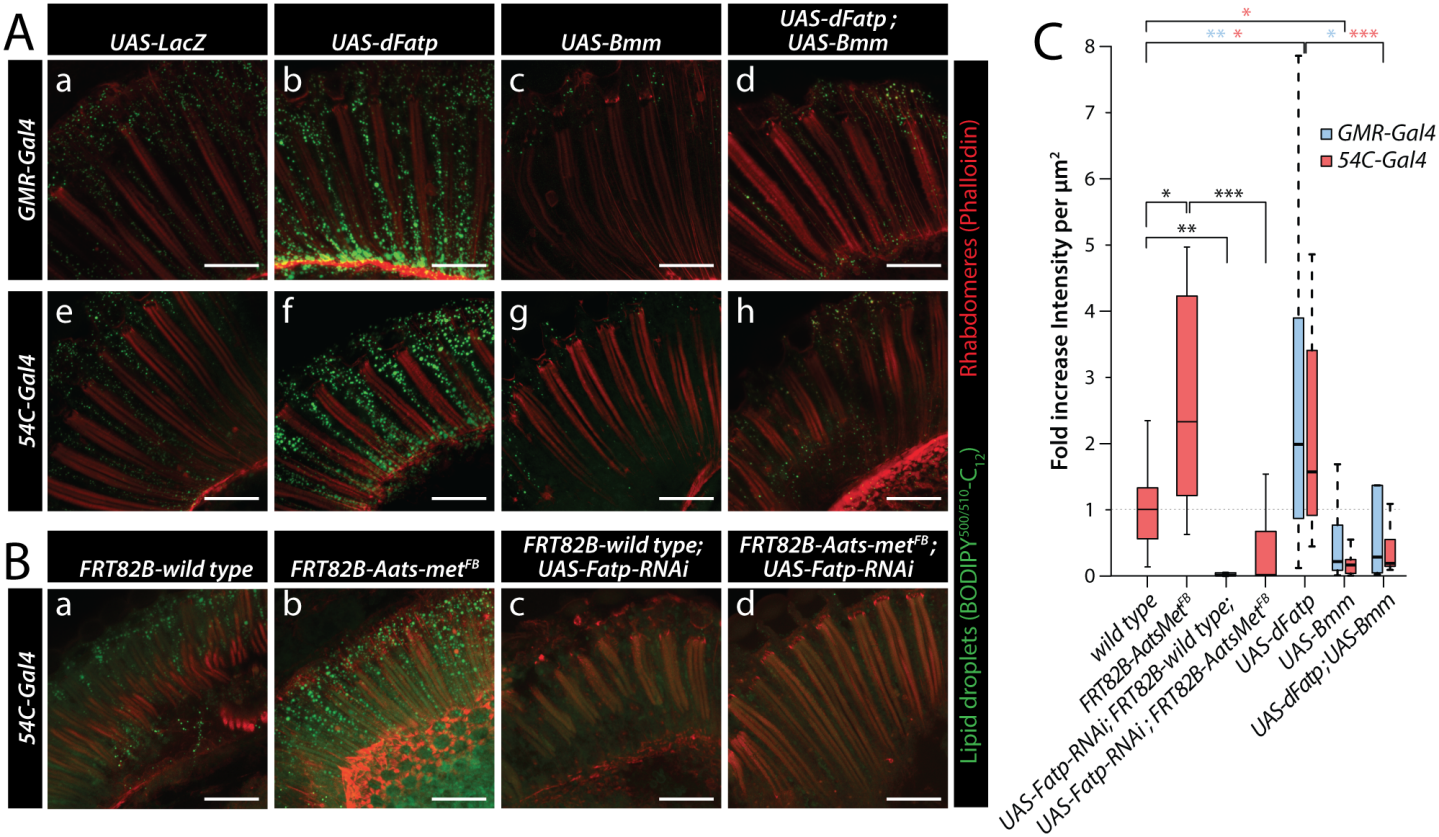
*dFatp* is required for lipid storage in *Drosophila* retina. (**A**) LD labeled with BODIPY^500/510^-C_12_ (green) were revealed using confocal microscopy in horizontal sections of whole mount retinas from one-day-old flies expressing *UAS-LacZ* (control), *UAS-dFatp* and/or *UAS-Bmm-lipase* under the control of a pan-retinal (*GMR-Gal4*) (a–d) or a dRPC-specific (*54C-Gal4*) (e–h) driver. Photoreceptors were counterstained with phalloidin-rhodamine (red). (**B**) BODIPY^500/510^-C_12_ (green) uptake in horizontal sections of whole eye wild-type (FRT82B-wild-type) and mutant (FRT82B-Aats-met^FB^) clone generated with the GMR-hid/FLP-FRT technique [49] from one-day-old flies in the absence (a, b) or presence of dRPC-specific (driven by *54C-Gal4*) *UAS-dFatp*-RNAi^[GD16442]^ (c, d). Photoreceptors were counterstained with phalloidin-rhodamine (red). Scale bar, 25 μm. (**C**) Quantification of BODIPY^500/510^-C_12_ uptake into lipid droplets from the images shown in (A) and (B). Data are presented as the fold change in fluorescence intensity (dots/μm^2^) compared with the FRT82B-wild-type flies. The boxes represent the median and lower and upper quartiles, and the whiskers represent the 1.5 interquartile range. N = 6–41 retinas per condition. Blue and red statistical stars indicate significant differences between wild type and retina overexpressing *dFatp* or *Bmm* with *GMR-Gal4* or *54C-Gal4*, respectively. Log adjusted values: *p<0.05, **p<0.01, ***p<0.001 by Tukey’s HSD paired sample comparison test.

To investigate the role of *dFatp* in LD formation and expansion in WT flies and in flies overexpressing *dFatp* (using the pan-retinal *GMR-Gal4* driver), we first measured the uptake of BODIPY^500/510^-C_12_, a fluorescent long-chain FA analog. We observed an accumulation of LDs (visible as green foci) in the retina of 1-day old WT flies and this accumulation was greatly increased when *dFatp* was overexpressed (Fig 1Aa, 1Ab and 1C). Importantly, expression of *brummer*, a gene encoding a TAG lipase (referred to here as Bmm-lipase) [29], reduced BODIPY^500/510^-C_12_ accumulation further demonstrating the lipid nature of the detected droplets in both WT and *dFatp*-overexpression conditions (Fig 1Ac, 1Ad and 1C). To overexpress *dFatp*, we also used the *54C-Gal4* driver, which was previously used as RPCs specific driver in several publications [9, 24, 27]. *dFatp* overexpression with the *54C* driver induced an important accumulation of LDs similar to the *GMR* driver (Fig 1Ae, 1Af and 1C). We verified the expression pattern of the *54C* driver (*54C-Gal4*, *UAS-GFP*) and confirmed that *54C* driver is strongly expressed in dRPCs (primary, secondary and tertiary pigment cells), although low levels of GFP expression were also observed in one or two photoreceptors per ommatidium, rarely in cone cells and not in bristle cells at 42h after puparium formation (APF) (S2A and S2B Fig). The developmental expression of the *54C* driver suggests that overexpression of *dFatp* during pupal development could account for the observed accumulation of LDs in adult fly retina. To confirm that the accumulation of LDs induced by *54C*-dependent overexpression of *dFatp* is truly RPC specific, we concomitantly expressed *Gal80* the inhibitor of *Gal4* with *Repo*, a pan-glial driver that is expressed in RPCs [30]. *Repo-Gal80* completely abolished the accumulation of BODIPY^493/503^ induced by the overexpression of *dFatp* with the *54C* driver (*54C-Gal4*, *UAS-dFatp*) confirming that the induction of LD by *54C* is RPC-specific (S2C and S2D Fig). In this experiment, we used the lipophilic probe BODIPY^493/503^ on fixed tissue instead of the BODIPY^500/510^-C_12_ on live tissue. Because we see increased *dFatp*-induced BODIPY staining in both cases (compare Fig 1Ae and 1Af with S2Ca and S2Cb Fig), this shows that *dFatp* overexpression mediates both the active uptake of BODIPY^500/510^-C_12_ into LD (Fig 1A) and the increase total LD stores revealed with BODIPY^493/503^. Similar to the *GMR* driver, the accumulation of BODIPY^500/510^-C_12_, induced by overexpression of *dFatp* with the *54C* driver was suppressed by *Bmm-lipase* concomitant overexpression (Fig 1Ag, 1Ah and 1C). We finally examined if the accumulation of LD due to *dFatp* expression requires Vitamin A, a precursor of retinal that is important for *dFatp* function in the regulation of Rh1 in photoreceptors [28]. As previously shown, we observed that the Vitamin A-deficient diet rescued photoreceptor degeneration in *dFatp* whole eye mutant clone ([28] and S3A Fig), but that the accumulation of LD induced by *dFatp* did not require Vitamin A (S3B and S3C Fig). This result suggests *dFatp* functions in LD biogenesis and in Vitamin A-dependent regulation of Rh1 are two distinct processes.

We then examined the requirement of *dFatp* in LD accumulation in retina of WT and *Aats-met*^*FB*^ mutant flies, which accumulate LD due to high mitochondrial ROS levels. As previously observed [9], BODIPY^500/510^-C_12_ uptake into LDs was increased in the *Aats-met*^*FB*^ mutant compared with the WT retina (Fig 1Ba, 1Bb and 1C). Expression of *dFatp*-specific dsRNA (RNAi) with the *54C-Gal4* driver abolished BODIPY^500/510^-C_12_ uptake in the WT flies and strongly reduced it in the *Aats-met*^*FB*^ mutants (Fig 1Bc, 1Bd and 1C). Furthermore, BODIPY^500/510^-C_12_ staining was completely abolished in *dFatp* whole eye mutant clones, confirming the specificity of *dFatp* dsRNA and the role of *dFatp* in LD formation (S3D Fig). Collectively, these data demonstrate that *dFatp* is necessary for LD accumulation in the *Drosophila* retina under physiological and pathological conditions.

**Fig 2:**
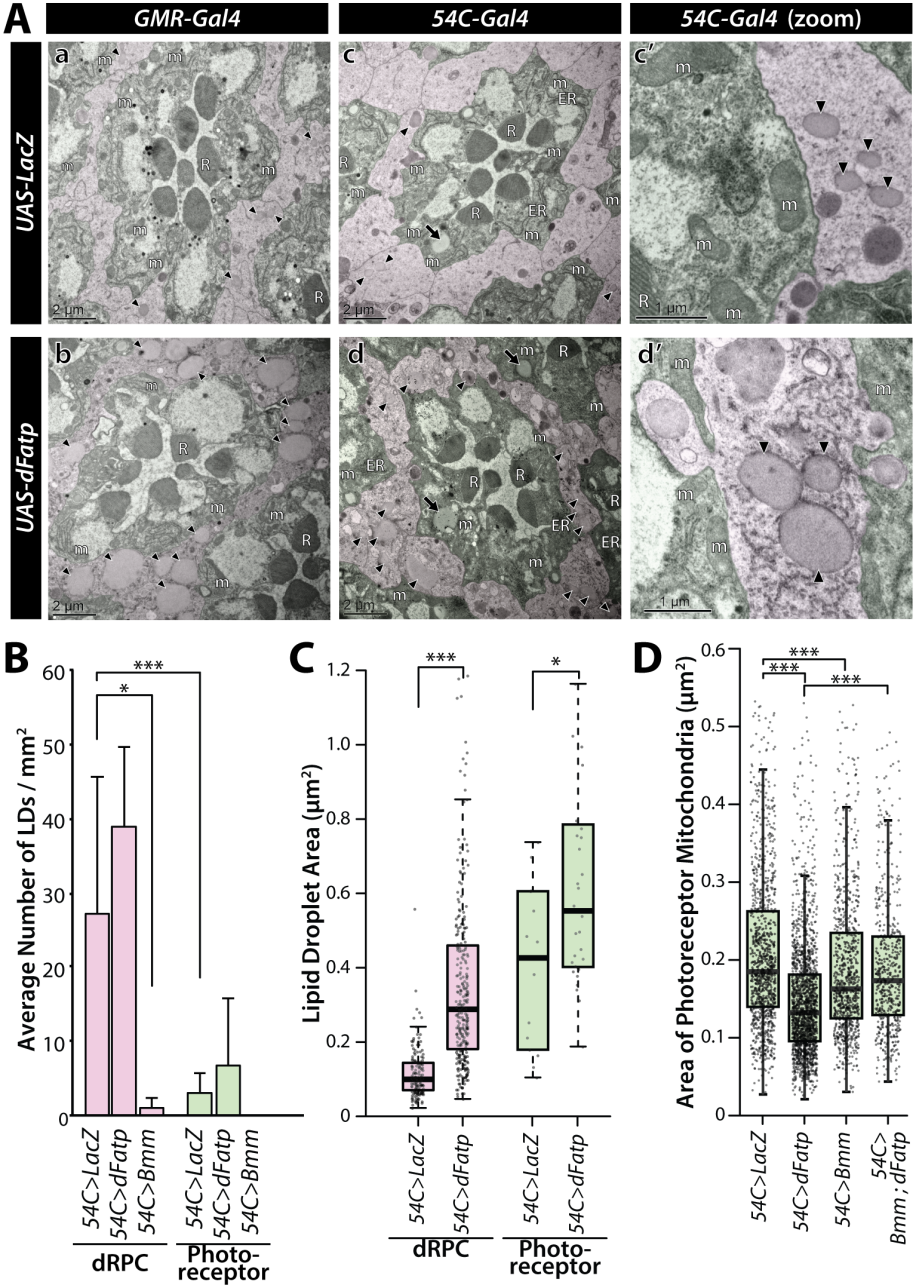
Lipid droplets are mainly localized in dRPCs and are increased by *dFatp* overexpression in *Drosophila* retinas. (**A**) TEM of ommatidia from one-day-old flies expressing *UAS-LacZ* (control) or *UAS-dFatp* under control of the pan-retinal (*GMR-Gal4*) or dRPC-specific (*54C-Gal4*) drivers. One ommatidium in each panel shows seven photoreceptors (false colored green) with central rhabdomeres surrounded by dRPCs (false colored pink). Lipid droplets are mainly located in dRPCs (black arrowheads) but can also be observed in photoreceptors (black arrows). Lipid droplet size was increased by *dFatp* overexpression (b, d, d’) compared to control conditions (a, c, c’). Scale bars, 2 μm (a–d), 1 μm (c’, d’). m, mitochondria; R rhabdomeres. (**B**) Quantification of lipid droplet density (number per surface area) in dRPCs and photoreceptors in flies with dRPC-specific expression (*54C*-*Gal4*) of *UAS-LacZ* (control), *UAS-dFatp*, or *UAS-Bmm-lipase*. Means ± SD of n = 4 eyes. (**C**) Quantification of lipid droplet size (area in m^2^) in dRPCs of flies with dRPC-specific (*54C*-*Gal4*) expression of *UAS-LacZ* (control) or *UAS-dFatp*. (**D**) Mitochondrial size (area in m^2^) in photoreceptors of flies with dRPC-specific expression of *UAS-LacZ* (control) or *UAS-dFatp*. The boxes represent the median and lower and upper quartiles, and the whiskers represent the 1.5 interquartile range. N = 4 to 5 flies, from which we analyzed >150 fields of view. Log adjusted values: *p<0.05, ***p<0.001 by Tukey’s HSD paired sample comparison tests.

To examine the subcellular localization of LDs in the retina, we performed transmission electron microscopy (TEM) of flies overexpressing *dFatp* using *GMR* or *54C* drivers. LDs were typically visible as homogeneous structures surrounded by a monolayer membrane in dRPCs and, to a much lesser extent, in photoreceptor cells (Fig 2A and 2B). Interestingly, *dFatp* overexpression significantly increased the size, but not the number, of LDs in both cell types (Fig 2A–2C). Finally, the expression *Bmm-lipase* with *54C-Gal4* caused a striking reduction in the number of LDs, not only in dRPCs but also in photoreceptors (Fig 2B and S4 Fig). This indicates that the monolayer membrane-encapsulated vesicles observed by TEM are indeed LDs.

The TEM images also indicated the expansion of LDs induced by *dFatp* overexpression concomitantly caused a significant decrease in the size of mitochondria in the photoreceptors (Fig 2D). We confirm that this reduction of mitochondrial size was correlated with LDs accumulation in a *dFatp*-dependent manner since co-expression of *dFatp* and *Bmm-lipase* prevented the decrease in photoreceptor mitochondrial size compared with expression of *dFatp* alone (Fig 2D). Notably, despite the reduction in size, the morphological integrity of mitochondria was preserved, suggesting that their function may remain intact and that accumulation of LD is not toxic to photoreceptors. Collectively, these results show modulating LD levels by increasing LD biogenesis (*54C>dFatp*) or LD lipolysis (*54C>Bmm*) in dRPCs affects LD and mitochondrial sizes in photoreceptor cells.

### Evidence of vesicle transfer from RPCs to photoreceptors

**Fig 3:**
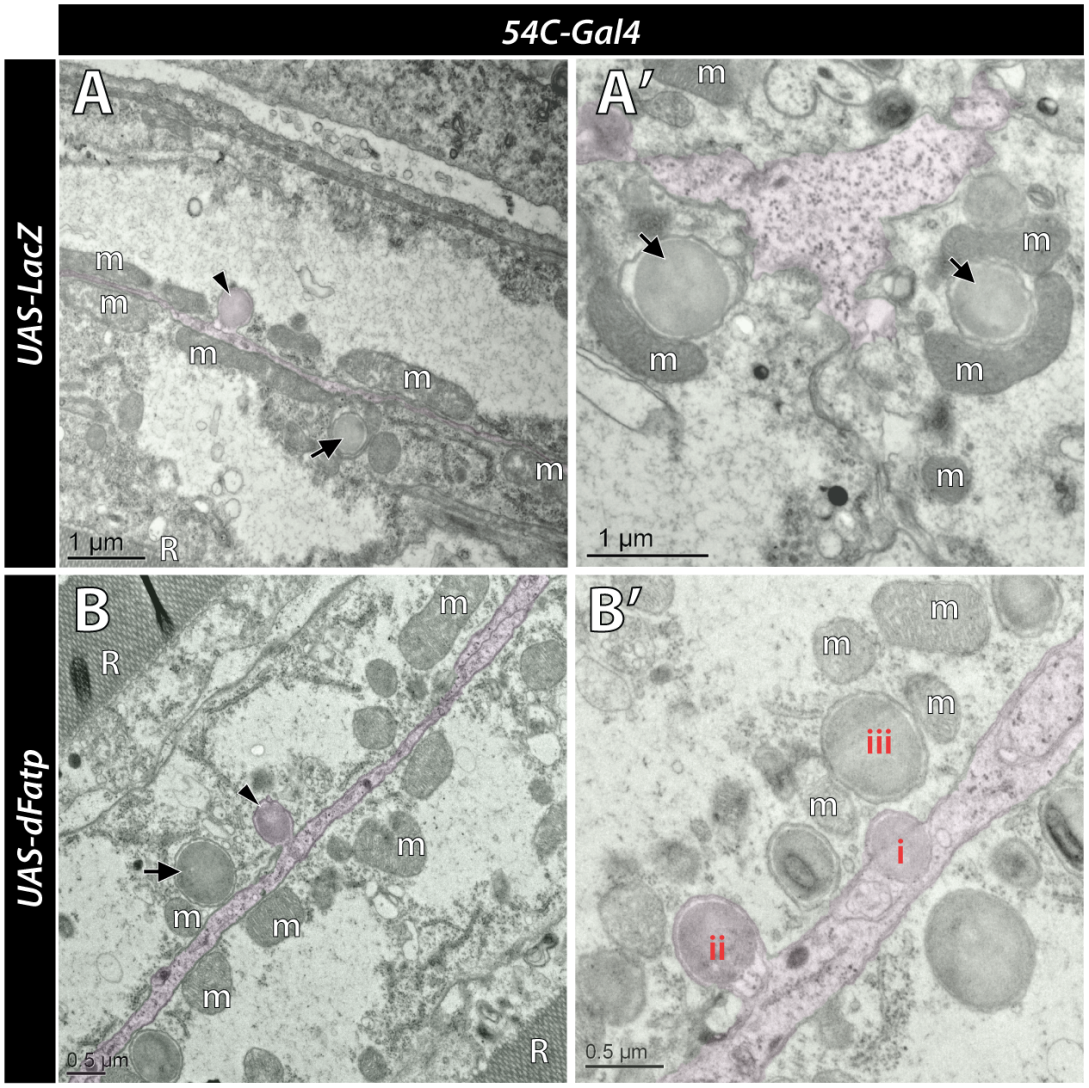
Transfer of vesicles from dRPCs to photoreceptors. Low (left) and high (right) magnification of retina tangential views visualized by TEM in one-day-old flies expressing (**A and A’**) *UAS-LacZ* or (**B and B’**) *UAS*-*dFatp* under the control of *54C-Gal4* driver. A dRPC (false colored pink) is visible sandwiched between two photoreceptors (false colored green). Arrowheads indicate lipid droplet-like vesicles entering an endocytotic invagination in the photoreceptor plasma membrane. Arrows indicate vesicles within photoreceptors surrounded by a double membrane. **(A’)** shows vesicles in close association with mitochondria (m) in a photoreceptor. **(B’)** shows various stages of the vesicle transfer from dRPC to photoreceptor. A vesicle within the dRPC (i) is taken up by the photoreceptor (ii), and surrounded by a double membrane once internalized within the photoreceptor (iii). Note the position of mitochondria (m) close to the vesicle. Scale bars, 1 μm (A and A’) and 0.5 μm (B and B’).

TEM images of retina showed various stages suggestive of the transfer of vesicles from dRPCs to photoreceptors in both wild-type and retina overexpressing *dFatp* with the *54C* driver. Vesicles can be seen within the dRPCs (i), in the process of being internalized into the photoreceptor by a process resembling endocytosis (ii), and fully internalized into the photoreceptor, where they appear surrounded by a double membrane (iii) (Fig 3). These vesicles were comparable in size to LDs (~0.25 μm^2^) but appeared slightly more electron dense. Due to their double membrane and localization at the proximity of the plasma membrane, these vesicles were distinguished from LD present in the cytoplasm of photoreceptors. These vesicles were seen juxtaposed to one or more mitochondria, which suggests that they contribute to or benefit from mitochondrial energetic function (Fig 3A and 3A’). These results suggest that the non-autonomous transfer of material contributes to the relay of information between dRPCs and photoreceptors.

### Expression of *hFATP1* in mRPC increases lipid storage and energy metabolism in the mouse retina

**Fig 4:**
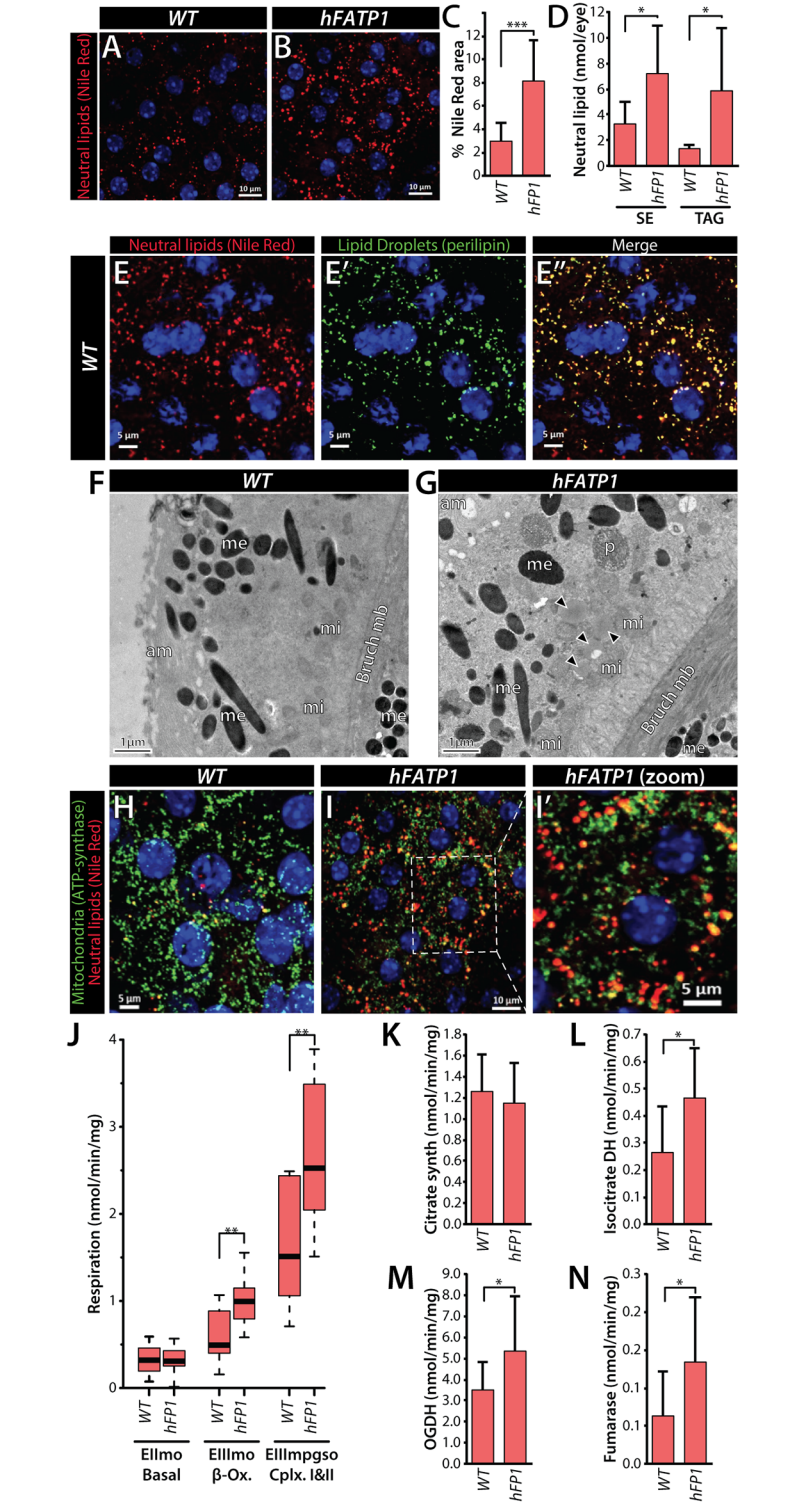
*hFATP1* overexpression in mouse RPCs increases neutral lipid accumulation and mitochondrial respiration. (**A, B**) Nile Red staining of neutral lipids (red) in mRPCs of a flat-mounted eye cup from wild-type (C57BL/6J) mice (**A**) and *hFATP1* transgenic mice (**B**). Nuclei are stained with DAPI (blue). Scale bars, 10 μm. (**C**) Quantification of Nile Red staining in wild-type (WT) and *hFATP1* transgenic (hFP1) mRPCs (as shown in A and B). Mean ± SD of n = 19 and 20 animals, respectively. (**D**) Quantification of sterol esters (SE) and triacylglycerides (TAG) in mRPCs from WT and *hFATP1* transgenic mice. Mean ± SD of n = 5 and 7 animals, respectively. (**E–E”**) mRPCs in whole-mount retinas from WT mice double-stained with Nile Red (**E**) and anti-perilipin (green, **E’**). The merged image (**E”**) shows extensive overlap between Nile Red and perilipin stainings. Scale bars, 5 μm. (**F, G**) TEM images of mRPCs from wild-type (C57BL/6J) mice (**F**) and *hFATP1* transgenic mice (**G**). am: apical membrane, Bruch mb: Bruch membrane, me: melanosome, mi: mitochondria, p: phagosome. (**H–I’**) mRPCs in whole-mount retinas of WT (**H**) and *hFATP1* transgenic (**I, I’**) mice double-stained with Nile Red and anti-ATP synthase antibody (green, localized to mitochondria). (**I’**) Magnification of the box in (**I**) shows the juxtaposition of mitochondria and neutral lipid stores. Scale bars, 5 μm (**H, I’**), 10 μm (**I**). (**J**) Quantification of mitochondrial respiratory function (O_2_ consumption) in RPCs isolated from WT and *hFATP1* transgenic mice. EIImo, basal respiratory rate; EIIImo, β-oxidation, EIIIMPGSO, global respiratory chain function of mitochondrial complexes I and II. The boxes represent the median and lower and upper quartiles, and the whiskers represent the 1.5 interquartile range. N = 21 and 10 WT and transgenic animals, respectively. (**K–N**) Activities of the TCA cycle enzymes, citrate synthase (**K**), isocitrate dehydrogenase (DH) (**L**), oxoglutarate dehydrogenase (OGDH) (**M**), and fumarase (**N**) in RPCs isolated from WT and *hFATP1* transgenic mice. Mean ± SD of n > 10 mice for each condition. *p<0.05, **p<0.01, ***p<0.001 for WT vs transgenic groups by two-sample t-test.

To determine whether the function of *dFatp* is conserved, we first asked whether the loss of photoreceptor cells observed in *dFatp*^-/-^ mutant retinas [28], could be rescued by the expression of the human homolog *hFATP1* in *Drosophila*. Indeed, photoreceptor-specific expression of *hFATP1* (driven by the rhodopsin 1 [*Rh1*] promoter) strongly reduced the loss of photoreceptors observed in *dFatp*^-/-^ mutant retinas (S5 Fig). This result supports the conservation of *hFATP1* and *dFatp* function in retinal homeostasis. To investigate the role of *hFATP1* in lipid storage in the mammalian retina, we employed our previously described transgenic mice, in which *hFATP1* is overexpressed using the mammalian RPC-specific VDM2 promoter (referred to as *hFATP1TG* mice) [31]. LDs in the retinas of WT (C57BL/6J) and *hFATP1TG* (Fig 4A and 4B) mice were detected by staining of neutral lipids with Nile Red. Notably, transgenic expression of *hFATP1* significantly increased neutral lipid staining, visible as red foci, in the mRPCs of *hFATP1TG* mice compared with WT mice (Fig 4A–4C). Moreover, lipid profiling of dissected mRPCs by gas-chromatography revealed that levels of sterol esters and TAGs, both of which are components of LDs, were higher in the *hFATP1* mRPCs compared with WT mRPCs (Fig 4D). We also observed that Nile Red staining largely co-localized with perilipin, a protein associated with LD membranes (Fig 4E–4E”), supporting the notion that the Nile Red-stained foci were indeed neutral lipid-containing LDs. The presence of LDs was also observed in mRPCs of *hFATP1TG* mice by TEM (Fig 4F and 4G). Taken together, these data indicate that *hFATP1* expression in mouse RPCs leads to accumulation of LDs, consistent with our findings in *Drosophila* RPCs.

Immunofluorescence staining for the mitochondrial marker ATP synthase revealed that LDs and mitochondria were in close proximity in the RPCs of both WT and *hFATP1TG* mice (Fig 4H–4I’), suggesting the possibility that LDs serve as an energy source in mRPCs. To test this hypothesis, we measured the mitochondrial respiratory rate in permeabilized mRPCs by monitoring O_2_ consumption after activation of different pathways (β-oxidation and Cx I + II). We found that *hFATP1* increased all respirations tested after addition of ADP to stimulate ATP production (EIIImo β-ox and EIIImpgso CxI+CxII) (Fig 4J). Consistent with this, the activities of the TCA cycle enzymes isocitrate dehydrogenase, 2-oxoglurarate dehydrogenase (OGDH), and fumarase were all significantly increased in retinal extracts from *hFATP1TG* mice compared with WT mice (Fig 4L–4N). This increased respiration rate in transgenic mRPCs is unlikely to be due to an increase in mitochondrial mass because the activity of citrate synthase was unaffected by *hFATP1* overexpression (Fig 4K). Taken together, these data demonstrate that *hFATP1* overexpression in mRPCs increases the size of LDs and suggests that they are substrates for energy production in the mitochondria.

**Fig 5:**
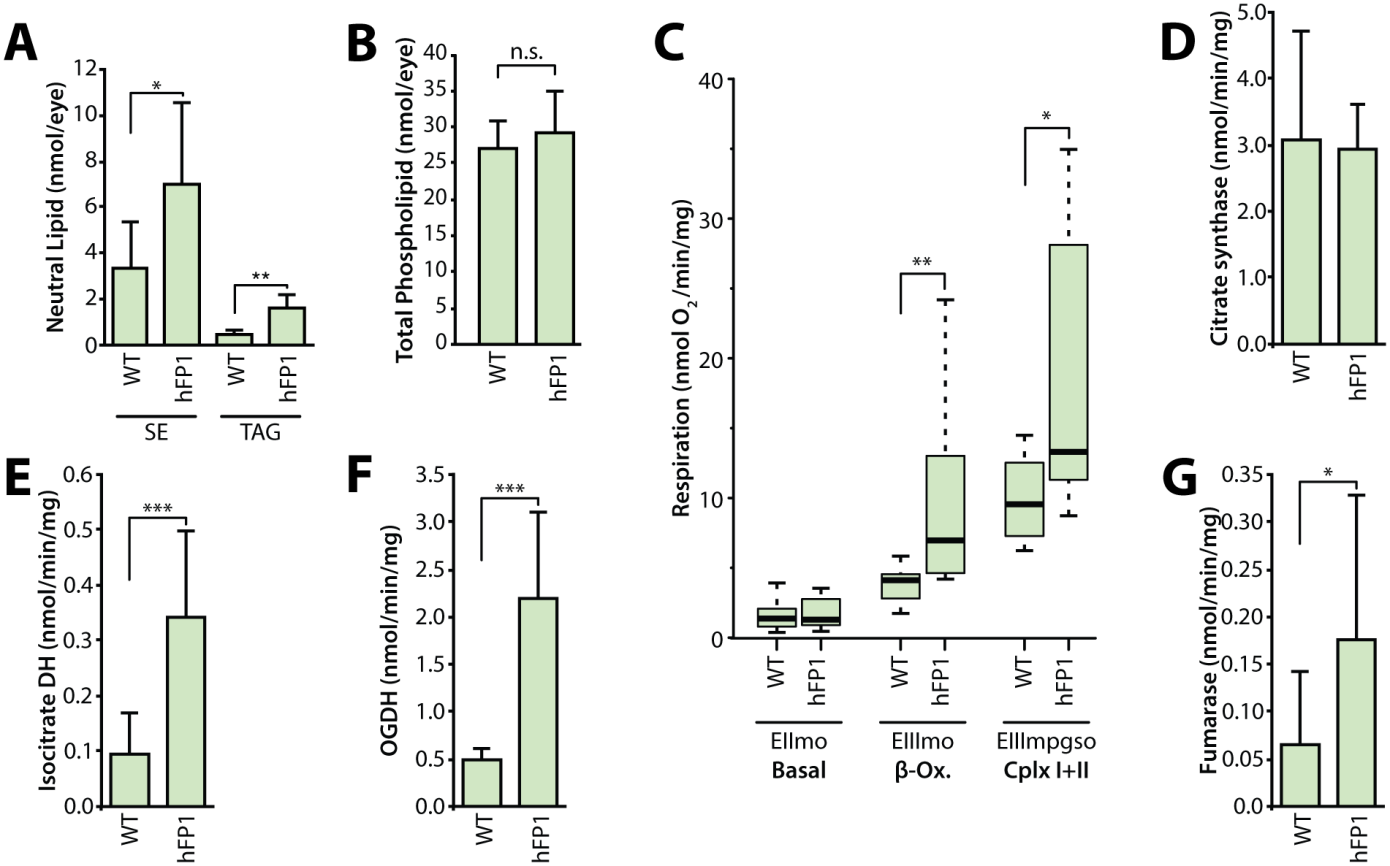
Cell non-autonomous effect of mRPC-specific *hFATP1* overexpression on the lipid content and respiratory rate in the neural retina. (**A**) Quantification of sterol esters (SE) and triacylglycerides (TAG) in the neural retina of 3-month-old WT and *hFATP1* transgenic mice. (**B**) Quantification of total phospholipid content in the neural retina of WT and *hFATP1* transgenic mice. Mean ± SD of n = 5 and 7 retinas, respectively. (**C**) Quantification of mitochondrial respiratory function in the neural retina of WT and *hFATP1* transgenic mice. EIImo, basal respiratory rate; EIIImbgso, β-oxidation; EIIImpgso, global respiratory chain function of complexes I and II. The boxes represent the median and lower and upper quartiles, and the whiskers represent the 1.5 interquartile range. N = 12 and 14 WT and transgenic retinas, respectively. (**D–G**) Activities of citrate synthase (**D**), isocitrate dehydrogenase (DH) (**E**), oxoglutarate dehydrogenase (OGDH) (**F**), and fumarase (**G**) in neural retinal extracts from WT and *hFATP1* transgenic mice. Mean ± SD of n > 9 retinas. *p<0.05, **p<0.01, ***p<0.001 for WT vs transgenic groups by two-sample t-test.

Next, we asked whether *hFATP1* expression and enhanced lipid storage in mRPCs could non-autonomously affect the physiology of juxtaposed neural retinal cells, which includes photoreceptor cells. mRPCs and the neural retina can be dissociated during dissection, which enables the effects of mRPC-specific *hFATP1* expression on the isolated photoreceptor cells to be examined. Lipid profiling of the neural retina revealed that sterol ester and TAG levels were higher while phospholipids levels remained unchanged in the neural retina of transgenic mice compared with WT mice (Fig 5A and 5B). Moreover, the magnitude of the increase was similar to that seen in isolated mRPCs (compare Fig 5A with Fig 4D). These results suggest a non-autonomous effect of mRPC-specific *hFATP1* expression similar to the observed phenotype in *Drosophila*. Next, analyses of the mitochondrial respiratory rate and TCA cycle enzyme activities in neural retinas showed a similar enhancement of O_2_ consumption (Fig 5C) and enzyme activities (Fig 5D–5G) in tissue isolated from *hFATP1TG* mice compared with WT mice. Taken together, these results indicate that *hFATP1* expressed in mRPCs not only increased lipid storage and mitochondrial respiration in the mRPCs themselves, but also in the neural retinal cells.

### Genetic modifications of LD accumulation perturb retinal homeostasis

**Fig 6:**
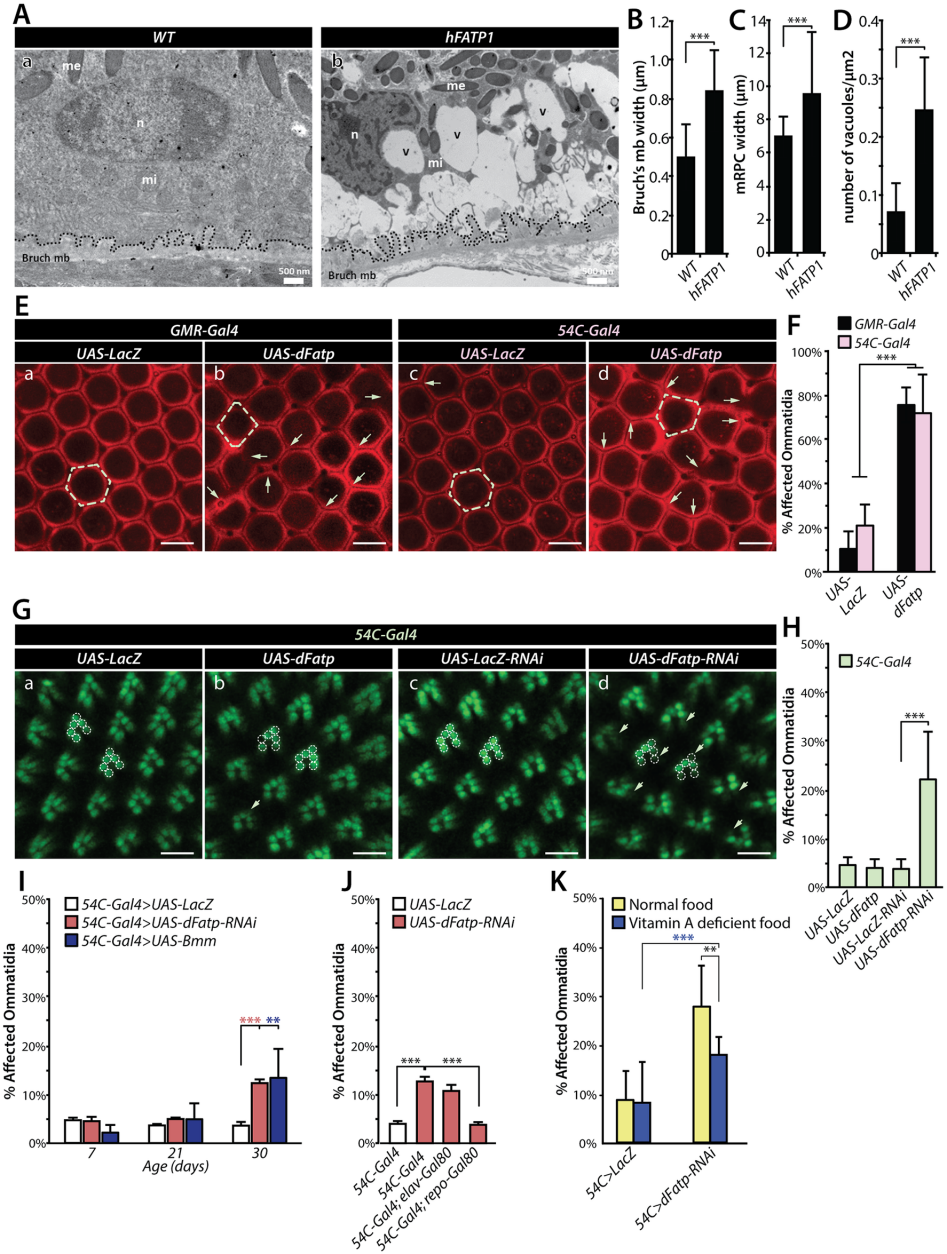
Consequences of *FATP*-dependent accumulation of LDs on RPCs and photoreceptors in *Drosophila* and mice. (**A**) TEM images of horizontal sections from 3-month-old wild-type (C57BL/6J) and *hFATP1* transgenic mice. Abundant vacuoles (v) and thickened Bruch’s membrane (mb, dashed lines) are evident in the aged *hFATP1* mice. me: melanosome, mi: mitochondria, n: nucleus. Scale bars, 500 nm. (**B–D**) Quantification of Bruch’s membrane width (**B**), mRPC width (**C**), and vacuole density in mRPCs (**D**) from the images shown in (A). Mean ± SD of n = 14 and 18 WT and transgenic animals, respectively. (**E**) Cornea neutralization method of the retinas using confocal fluorescence microscopy (dRPC autofluorescence) of 30-day-old *Drosophila* expressing (a, c) *UAS-LacZ* (control) or (b, d) *UAS-dFatp* throughout the retina (*GMR-Gal4*) or in dRPCs alone (*54C-Gal4*). Arrows show loss of dRPCs resulting in disorganization and loss of typical hexagonal ommatidial shape (dashed outline) upon *dFatp* overexpression. Scale bars, 10 μm. (**F**) Quantification of ommatidia with missing dRPCs (affected ommatidia) in retinas shown in (E). Mean ± SD of n = 5 and 8 control and transgenic flies, respectively. Black bars, *GMR-Gal4*; pink bars, *54C-Gal4*. (**G**) Visualization of retinas of 40-day-old Rh1-GFP-expressing flies by the cornea neutralization method with dRPC-specific expression of (a) *UAS-LacZ* (control), (b) *UAS-dFatp*, (c) *UAS-LacZ* RNAi (control-RNAi), or (d) *UAS-dFatp*-specific RNAi (*dFatp*-RNAi^[GD9406]^). (b) *dFatp* overexpression in dRPCs does not affect photoreceptor survival, as indicated by intact rhabdomeres (dashed outlines). (d) *dFatp*-RNAi^[GD9406]^ expression induces the loss of rhabdomeres (arrows). Scale bars, 10 μm. (**H**) Quantification of ommatidia with one or several missing photoreceptors (affected ommatidia), as shown in (G). (**I**) Quantification of affected ommatidia with missing photoreceptors in 7, 21 and 30 day-old flies expressing *UAS-LacZ*, *UAS-dFatp* or *UAS-Bmm* under the control of *54C-Gal4*. (**J**) Quantification of affected ommatidia with missing photoreceptors in 30 day-old flies expressing *UAS-LacZ*, or *UAS-dFatp* under the control of *54C-Gal4* combined with *repo-Gal80* (white bar), *elav-Gal80* (pink bar) or wild-type (green bar). (**K**) Quantification of affected ommatidia with missing photoreceptors in 30 day-old flies expressing *UAS-LacZ* or *UAS-dFatp-RNAi* under the control of *54C-Gal4* in normal (yellow) or Vitamin A deficient (blue) diet. Mean ± SD of n = 5–7 flies/condition. *p<0.05, **p<0.01, ***p<0.001 by two-sample t-test.

We next examined the consequences of LD accumulation caused by overexpression of *FATP* for the viability of RPCs in mice and *Drosophila*. TEM analyses showed that mRPC-specific expression of *hFATP1* induced vacuolization of the mRPCs and thickening of both mRPC and Bruch’s membranes in 3-month-old mice (Fig 6A–6D). Similarly, pan-retinal or dRPC-specific expression of *dFatp* caused a moderate loss of dRPCs (one to two missing dRPCs in most ommatidia), resulting in perturbation of the ommatidia organization in *Drosophila* retina (Fig 6E and 6F). In contrast to dRPCs, photoreceptor cell survival (visualized by expression of Rh1-GFP) was unaffected by *dFatp* overexpression or by the knockdown of the *Bmm-lipase* which induced the accumulation BODIPY^493/503^ similar to *dFatp* overexpression (Fig 6G and 6H and S6 Fig). These results in *Drosophila* retina are in agreement with our previous findings in mouse retina showing that photoreceptor layers were unaffected when *hFATP1* was overexpressed in mRPCs [31]. Therefore, *FATP*-mediated LD accumulation in RPCs does not appear to be deleterious to photoreceptor cells under physiological conditions.

In contrast, knockdown of *dFatp* with the *54C* driver, which is associated with a diminution of LD content, resulted in late onset photoreceptor degeneration in the *Drosophila* retina (Fig 6Gc, 6Gd and 6H). Importantly, this photoreceptor degeneration induced by expression of *dFatp-RNAi* with the *54C* driver was suppressed by Gal80 expression in dRPCs with the Repo driver (Fig 6J). In contrast, blocking the expression of *dFatp-RNAi* specifically in photoreceptors (elav-Gal80) did not inhibit photoreceptor degeneration (Fig 6J). This supports the fact that *dFatp*-dependent accumulation of LD in dRPC is non-autonomously required for photoreceptor viability. In agreement with the idea that LD are required for photoreceptor viability, we also observed a late onset photoreceptor degeneration similar to *dFatp* knockdown, by forcing LD lipolysis by overexpressing the *Bmm-lipase* or inhibiting LD biogenesis by expressing a RNAi against *midway* (*mdy*), a diacylglycerol acyltransferase (DGAT) that functions in the formation of TAG (Fig 6I and S6 Fig).

Next, we examined the effect of Vitamin A-deficient diet on photoreceptor degeneration induced by *54C>dFatp-RNAi*. As previously shown [28], the Vitamin A deficient diet strongly suppressed photoreceptor degeneration in *dFatp* mutant (S3A Fig). In contrast, we only observed a modest rescue of photoreceptor degeneration in flies expressing *dFatp-RNAi* in dRPCs with this diet supporting the idea that a Vitamin A independent dRPC role of *dFatp* in photoreceptor degeneration is predominant (Fig 6K). Collectively, these results indicate that *dFatp*-dependent LDs support photoreceptors viability during the normal aging process.

**Fig 7:**
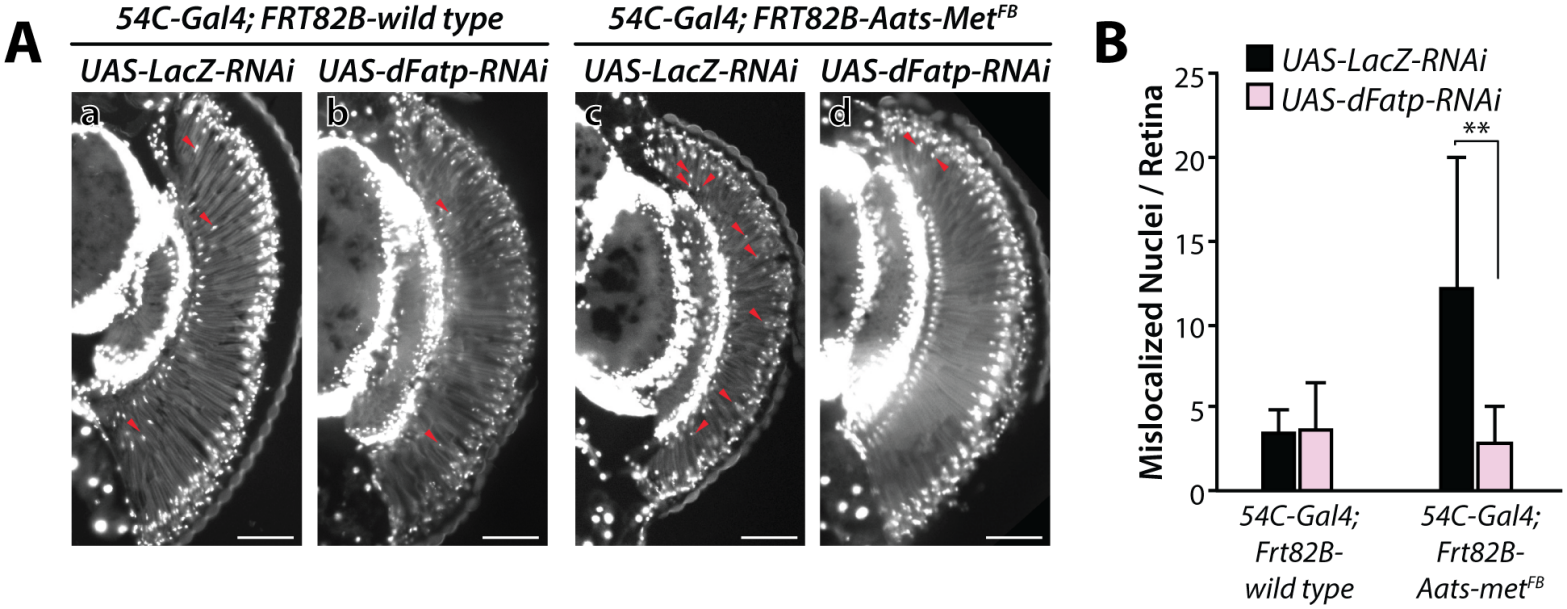
dRPC-specific knockdown of *dFatp* suppresses Aats-met^FB^-induced photoreceptor degeneration in *Drosophila* retina. (**A**) Epifluorescence microscopy of retinal sections on whole eye clones generated with the GMR-hid/FLP-FRT technique [49] from 5-day-old FRT82B-wild-type (a, b, control) or FRT82B-*Aats-met*^*FB*^ mutant (c, d) flies expressing *UAS-dFatp-RNAi*^*[GD16442]*^ (b, d) or *UAS-Lac-RNAi* (a, c) under the control of *54C-Gal4*. In *Aats-met*^*FB*^ mutant (c), nuclei (stained with DAPI, white) are mislocalized between the distal and proximal part of the retina (red arrows), indicative of dying photoreceptors. dRPC-specific expression of *UAS-dFatp*-RNAi^[GD16442]^ markedly reduces the number of dying photoreceptors in *Aats-met*^*FB*^ mutant (d). Scale bars, 100 μm. (**B**) Quantification of mislocalized nuclei, as shown in A. Mean ± SD of n > 6 retinas. **p<0.01 by two-sample t-test.

Finally, we asked whether LD accumulation in dRPCs was beneficial or deleterious to photoreceptors under stress conditions. For this, we examined young (5-day-old) *Aats-met*^*FB*^ mutant flies, in which *dFatp* RNAi prevents LD accumulation (see Fig 1B and 1C). Importantly, *dFatp* RNAi alone does not induce photoreceptor degeneration in 5-day-old flies [28], but the mutation *Aats-met*^*FB*^ itself causes some degeneration, allowing us to examine the consequence of *dFatp* RNAi in *Aats-met*^*FB*^ mutant on photoreceptor survival. We analyzed the apical/distal position of photoreceptor nuclei, which are normally clustered apically in the WT retina but are mislocalized under conditions associated with photoreceptor death [28]. Monitoring of mislocalized nuclei is a useful readout of cell death in situations where photoreceptor rhabdomeres are difficult to count due to their rapid degradation, as is the case for *Aats-met*^*FB*^ mutants. We observed an increase in the number of nuclei mislocalized between the proximal and distal part of the retina in *Aats-met*^*FB*^ mutants compared with WT flies (Fig 7Aa, 7Ac and 7B), consistent with previous reports for this mutant [9]. However, the mislocalization was completely reversed by dRPC-specific *dFatp* RNAi (Fig 7Ad and 7B). These results indicate that *FATP*-mediated LD accumulation in dRPCs is harmful to photoreceptors under conditions of pathological oxidative stress (*Aats-met*^*FB*^ mutants), in stark contrast to the beneficial effect under physiological conditions.

## Discussion

Here, we studied the consequences of dysregulated lipid storage in RPCs on photoreceptor structure and function in both *Drosophila* and mouse retinas. Our results show that the *FATP* dependent-mechanisms of lipid storage and communication between RPC and photoreceptor layers are largely conserved in flies and mice. We made several novel observations in this study. We showed that *FATP* expression is required for LD formation in mouse and *Drosophila* RPCs. Interestingly, FATP has an acyl-CoA synthetase activity that is thought to facilitate cellular uptake of FAs (reviewed in [16]). FATP also interacts with DGAT-2 on the endoplasmic reticulum, where it has been show to function in LD expansion in *C*. *elegans* [13]. The fact that *dFatp* overexpression increases LD size but not number also suggests that the role of *FATP* in LD expansion is conserved in *Drosophila*. Interestingly, retinal LD accumulation induced by *FATP* overexpression resembles one of the key hallmarks of AMD pathology; that is, the accumulation of lipids in RPCs and of drusen in Bruch’s membrane [32]. We also found that mRPC-specific *hFATP1* expression increased non-autonomously neutral lipids, β-oxidation, TCA enzyme activity, and mitochondrial respiration in photoreceptors. The fact that overexpression of *FATP* promotes both catabolic (mitochondrial respiration, TCA cycle, β-oxidation) and anabolic (increase of TAG and SE) functions that are distinct processes could be interpreted as followed. First, *FATP* promotes the import of fatty acids, which are used for different metabolic function including catabolism and anabolism within the cell. And second, FATP carries an acyl-coA synthase activity that is central in lipid metabolism for lipid elongation, unsaturation but also β-oxidation [16]. In flies, we did not evaluate the mitochondrial respiration rate due to the technical difficulty of dissociating RPCs from photoreceptor cells. However, mitochondria were intact by TEM and frequently observed juxtaposed to LDs in both flies and mice. The proximity between LD and mitochondria suggests that release of TAGs from LDs mediated by lipases could provide an energy source for the mitochondria. This hypothesis is supported by studies showing that physical proximity between LDs and mitochondria is important for β-oxidation of FAs [33].

In flies, we used the *54C* driver, which is strongly expressed in dRPCs to examine the non-autonomous role of *dFatp* on adjacent photoreceptor as previously described [27]. Because the *54C* driver also showed a low level of expression in few photoreceptors during pupal development, we took advantage of the Gal80 system to show that photoreceptor degeneration observed in flies carrying *54C>dFatp-RNAi* is due to the expression *of dFatp-RNAi* in dRPCs but not in photoreceptors. These results support a non-autonomous role of *dFatp* in fly RPCs as it is observed in the mouse. Altogether, we have shown that both the function of *FATP* in RPCs and its effect on photoreceptor cells are largely conserved between flies and mammals.

Although our data demonstrate that *FATP*-mediated LD accumulation in RPCs provides a metabolic signal to photoreceptors, it is not clear whether the signal itself is a lipid or another signaling molecule. Recent work proposed that in mutants that carry dysfunctional mitochondria, the accumulation of LDs in RPCs requires transfer of lactate from RPCs to photoreceptors [27]. The increased lactate levels in photoreceptors enhanced the activity of the TCA cycle and synthesis of FAs, which then underwent apolipoprotein-mediated transfer back to RPCs and induced LD formation [27]. A similar mechanism was described in mouse neuronal/glial co-cultures, raising the possibility that it may also be operating in the retina of *hFATP1TG* mice [27]. Our ultrastructural analysis supports the possibility of an exchange of electron dense material between RPCs and photoreceptor cells in *Drosophila*. Indeed, electron dense vesicles—similar in appearance and size to LDs—were observed in contact with dRPCs, as invaginations in the photoreceptor membrane, and finally as double-membraned structures in close proximity to the mitochondria in photoreceptors. Associations between LDs and mitochondria have also been observed in skeletal and heart muscle, where it was proposed to facilitate β-oxidation [34, 35, 33]. Although further studies will be required, our ultrastructural findings provide a hint that vesicle transfer could be a route of communication between RPCs and photoreceptors. Collectively, our results demonstrate that crosstalk between RPCs and photoreceptors is crucial for normal photoreceptor homeostasis and that its breakdown may contribute to retinal pathologies.

In our previous work, we showed that *dFatp* is expressed at higher levels in dRPCs than in photoreceptors, and that loss of *dFatp* led to a cell-autonomous photoreceptor degeneration [28]. We had proposed that degeneration of photoreceptors in the *dFatp* mutant is due to a failure to degrade Rh1, which accumulates to toxic levels and triggers apoptosis. Indeed, photoreceptor loss was rescued by down-regulating Rh1 levels in a Vitamin A-deficient diet [28]. We now show that photoreceptor undergo degeneration in flies expressing *dFatp*-RNAi in dRPCs even if fed in a Vitamin A-deficient diet. This indicates that *dFatp* expression in dRPCs also contributes to photoreceptor viability via its role in LD biosynthesis. This is further supported by our results showing that forcing LD lipolysis or inhibiting LD biogenesis induced late onset photoreceptor degeneration similar to *dFatp* knockdown. Therefore, *dFatp* would have two distinct functions in photoreceptors and dRPCs. In photoreceptors, *dFatp* is required for optimal Rh1 metabolism, presumably due to its role in phosphatidic acid synthesis and Rh1 trafficking [16, 36, 37]. In dRPCs, *dFatp* is required for expansion of LDs, which can then be metabolized for energy production. Finally, hFATP1 has been shown to directly interact with visual cycle enzymes in RPC to regulate retinyl-ester-dependent RPE65 isomerase necessary for production of 11-cis-retinal and rhodopsin [38, 39]. In Cubizolle et al., we have also shown by HPLC that retinyl esters accumulate in mRPC of transgenic mice expressing *hFATP1* [31]. We had proposed that the accumulation of retinyl ester could be due to either the inhibition of RPE65 or to an increase of long-chain fatty acids (LCFA) induced by *hFATP1* expression, which results in increased retinyl esters. Indeed, incorporated LCFA can form phosphatidyl choline (PC), which serves in the esterification of all-trans-retinol to form all-trans-retinyl esters in a reaction catalyzed by the Lecithin retinol transferase (LRAT) [40]. Together, our results show that in addition to the role of *FATP* in the regulation of visual cycle precursors in mouse RPC [31] and in the regulation of Rh1 levels in Drosophila photoreceptor [28], *FATP* has a conserved RPC-specific function in lipid storage that promotes the increase of neutral lipids in photoreceptors and the overall energy metabolism by the mitochondria in RPC and photoreceptor layers.

Our results show that the ectopic accumulation of LDs in RPCs induced by overexpression of *FATP* is not toxic to photoreceptors in either *Drosophila* (this study) or mice [31], and that physiological levels of LD are protective for *Drosophila* photoreceptors during aging. The loss of photoreceptors following depletion of LDs may therefore be due to the dwindling supply of energy, especially since photoreceptors have high-energy demands [5]. This hypothesis is supported by the proximity of LD and mitochondria that is observed in both mouse and fly retina that overexpress *FATP*. It is also supported by the increased mitochondrial respiration and β-oxidation rates measured in both RPC and PR layers of mice retina that overexpress *hFATP1*. Another non-exclusive hypothesis supported by recent reports, is that *de novo* LD biogenesis protects against lipotoxicity under conditions of low nutrient supply or high-energy demand as it was shown in adipocytes and mouse embryonic fibroblasts [41, 42]. Collectively, these results indicate that lipid storage in LDs is physiologically required for photoreceptor health during aging. In addition to acting as an energy source, LDs may also reduce the cellular sensitivity to ROS. The latter possibility is supported by the finding that ROS induced by light exposure, which promotes the accumulation of photo-damaged proteins and lipids [43], enhances photoreceptor degeneration to a greater extent in *dFatp* mutant flies than in WT flies [28]. Similarly, LD accumulation in glia protects against ROS-induced damage of the developing nervous system of *Drosophila* upon exposure to hypoxia [8]. Moreover, LDs are not uniform structures but exist in various forms with differing potential functions, such as storage of phospholipids, vitamin E, and cellular toxins [10].

**Fig 8:**
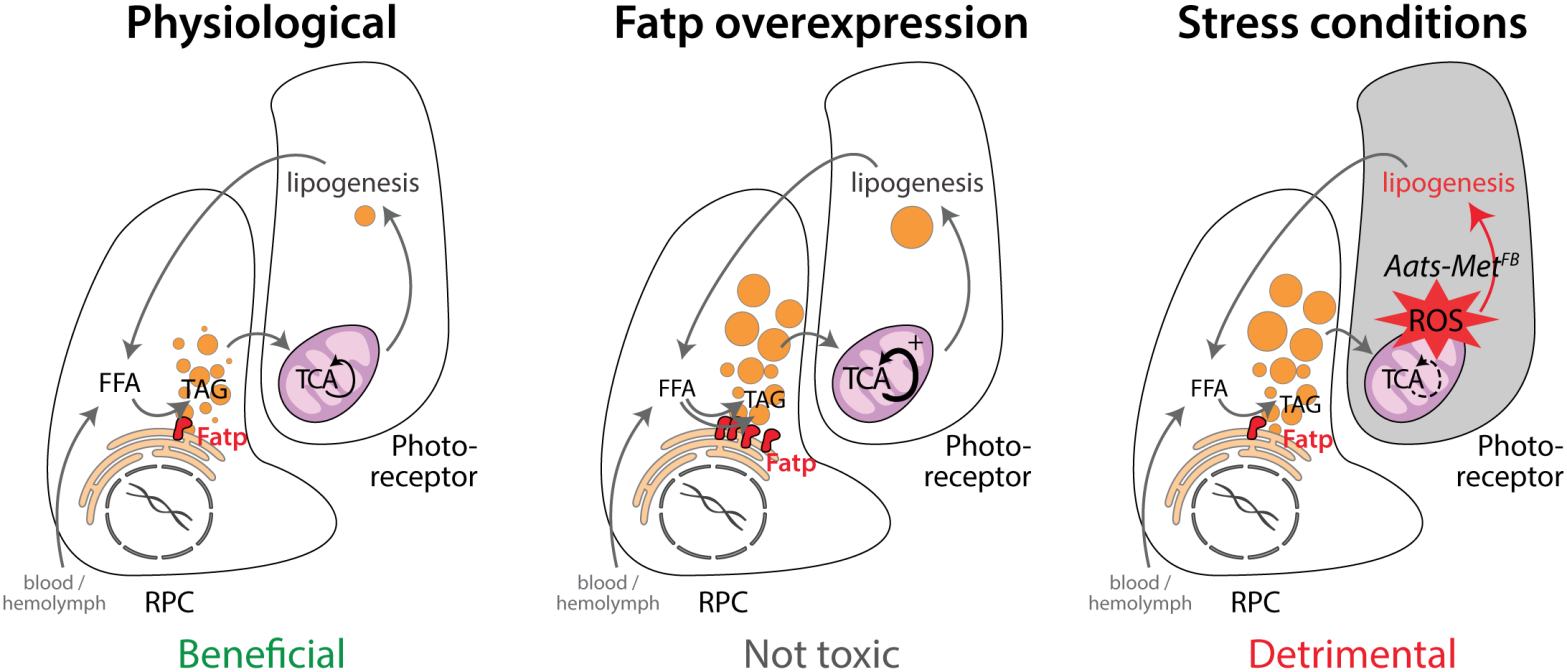
Schematic of the role of *FATP* in the metabolism of lipid droplets and communication between RPCs and photoreceptors under physiological and pathological conditions. Under physiological conditions, *FATP* is involved in the maintenance of LD and their presence in RPCs is required and beneficial for photoreceptor health. Indeed, the removal of LD by expression of *dFatp-RNAi*, *Mdy-RNAi* or *Bmm* induces a progressive photoreceptor degeneration in flies. Overexpression of *FATP* in RPCs expands the cellular LD content, which has a cell non-autonomous stimulatory effect on neighbouring photoreceptors that increases LD (in flies), neutral lipids, mitochondrial respiration and β-oxidation (in mice). LD accumulation in condition of overexpression of *FATP* in RPCs is not toxic for photoreceptors and could be considered beneficial through increased mitochondrial function and nutrient availability in these cells. Under conditions of oxidative stress (e.g., *Aats-met*^*FB*^ mutant flies, in which ROS levels are elevated in photoreceptors), LD are no longer beneficial. In this scenario, LD could be considered as detrimental which could be due to their abnormal turnover under high ROS leading to enhanced lipid peroxidation.

Our data establish that LDs are not toxic to retinal cells under physiological conditions, but that they can contribute to neurodegeneration under some stress conditions as shown here with the *Aats-met*^*FB*^ mutants (model in Fig 8). In these mutants, suppression of LD accumulation in dRPCs is neuroprotective, suggesting that the LDs non-autonomously promote photoreceptor cell death under conditions of oxidative stress. This is consistent with studies of flies with mitochondrial defects that cause high ROS levels in neurons, which demonstrated that removal of LDs by *dFatp* knockdown rescued retinal degeneration in *sicily* (*Drosophila* homolog of the human nuclear encoded mitochondrial gene NDUFAF6) and *marf* (*Drosophila* homolog of the mitochondrial fusion GTPases, Mitofusin 1 and 2) mutants and that ectopic expression of *Bmm-lipase* rescued photoreceptors in *Aats-met* mutants [9, 27]. One possible explanation as proposed by Liu et al. [27], is that high levels of ROS induce an abnormal initial raise followed by a decrease of LD as the pathology progresses. This perturbed turnover could override LD beneficial role and enhances lipid peroxidation and photoreceptor death. Collectively, our findings and those of others suggest that LDs may play a protective or beneficial role under physiological conditions and be deleterious under pathological conditions.

In conclusion, despite the difference in mouse and fly retinal architecture, RPCs appear to be functionally homologous in both species with respect to providing metabolic support for adjacent photoreceptors. *Drosophila* RPCs form a tight barrier between the hemolymph and photoreceptors, similar to the mammalian outer retina [44], implying that dRPCs, like mammalian RPCs, may play an active role in metabolite exchanges with photoreceptors [32]. Similar to dRPCs, it was recently shown that *Drosophila* cone cells, which are also accessory cells of photoreceptors, express glial cell markers and are important to maintain photoreceptor function in the adult fly [45]. Thus, combined with our data, these results support that both types of accessory cells- dRPCs and cone cells- carry glial functions similar to the support functions of Müller glia and retinal pigment epithelium (here mRPC) in the vertebrate retina. We propose that the RPC–photoreceptor interaction is a conserved paradigm between *Drosophila* and mammalian that will be a useful model to investigate mammalian RPC disorders, such as AMD, particularly given *Drosophila* genetic tractability.

## Material and methods

### Ethics statement

Experiments were carried out in accordance with the European Communities Council Directive of 24 November 1986 (86/609/EEC) and the French Ethical Committee (CEEA-LR-12141) guidelines for the care and use of animals for experimental procedures.

### Drosophila genetics

The fly stocks used in this study were: *54C-Gal4* (BS#27328) [24], *GMR-Gal4*, *Rh1-Gal4* [46], UAS-dcr2; *eyeless-Gal4 (ey-Gal4)*, *GMR-Gal4*, *Rh1-GFP* (gift from Claude Desplan), *UAS-dFatp*, *FRT40A dFatp*^*k10307*^ [28], *Rh1-Gal4*, *eyflp; FRT40A Rh1-TdTomatoNinaC; UAS-GFP* [47], *UAS-bmm* [29], *UAS-dFatp-RNAi* (VDRC stocks GD16442 and GD9604), *UAS-bmm-RNAi* (BS#25926); *UAS-Mdy-RNAi* (VDRC stock GD1749 #6367), *FRT82*^*B*^ *Aats-met*^*FB*^ (BS#39747), and *Rh1-GFP* [48]. *Repo-Gal80* and *Elav-Gal80* (gift from Laurent Seugnet, CRNL, Lyon, France). Mitotic whole eye mutant clones for *dFatp*^*k10307*^ and *Aats-met*^*FB*^ were generated using GMR-hid/FLP-FRT technique by crossing a mutant line carrying the FRT-containing chromosome with a line containing *ey-flp* and *FRT40A GMR-hid* (for *dFatp*^*k10307*^) or *FRT82B GMR-hid* (for *Aats-met*^*FB*^) [49]. Human *FATP1* cDNA, a gift from Celine Haby (IGBMC, Strasbourg), was cloned via BamHI and EcoRI into a pUAST-w+-attB transgenic vector. Transgenic lines were generated by Best Gene (Chino Hills, CA, USA) using PhiC31 integrase-mediated transgenesis [50] at the same site used for *dFatp* (65B2). Flies were maintained on standard corn medium at 25°C or on Vitamin A-deficient medium. Vitamin A-deficient medium contained yeast (12 g), agar (1,5 g), sucrose (7,5 g), cholesterol (0,03 g), sodium methyl-4-hydroxybenzoate (1.15M, 3.75 mL) and propionic acid (0.72 mL) in distilled water (150 mL) as described [28].

### Lipid droplet staining using BODIPY lipid probes

The retinas of 1-day-old flies were dissected in ice-cold HL3 medium [51] according to [28]. Tissues were then processed for staining with either C_1_-BODIPY^500/510^-C_12_ (D-3823) or BODIPY^493/503^ (D-3922; both acquired from Life Technologies) as stated in figure legends.

For BODIPY^500/510^-C_12_, dissected retinas were warmed to 25°C and directly incubated for 30 min with 1.5 μg/mL BODIPY^500/510^-C_12_ (D-3823), washed three times in HL3 medium, fixed in 4% paraformaldehyde (PFA, Electron Microscopy Sciences) for 15 min at room temperature (RT), washed three times in wash buffer (PBS containing 0.1% Triton-X100), and incubated for 16 h at 4°C in wash buffer supplemented with 25 ng/mL phalloidin-TRITC (Sigma).

For BODIPY^493/503^, dissected retinas were fixed first in 4% PFA for 15 min at RT, washed three times and then incubated with 75 ng/mL BODIPY^493/503^ and 25 ng/mL phalloidin-TRITC for 16 hours at 4°C.

Subsequently, samples were washed three times in wash buffer, mounted on a bridge slide in Vectashield (Vector Laboratories), and stored at −20°C until analysis. 16-bit image stacks were acquired on a Zeiss LSM800 microscope and processed for quantification using ImageJ2 software [52]. Images were filtered for noise using Gaussian Blur 3D (σ = 1), after which a Z-projection was made. LDs were identified by thresholding the images, and the integrated density of the signal and total retinal area were measured. The integrated density of BODIPY staining divided by the total area of the retina was normalized to the control for each experiment.

### Statistical analysis

Statistical analyses were performed using R. Group differences were analyzed by t-test or Tukey’s HSD paired sample comparison test, as specified in the figure legends.

### Imaging of photoreceptors and RPCs by the cornea neutralization method in living flies

Living flies, maintained for 30 days at 25°C on a 12h light/dark cycle, were anesthetized using CO_2_, embedded in 1% agarose covered with cold water [53], and imaged using a Leica SP5 upright confocal microscope. RPCs were visualized by pigment autofluorescence (excitation 514 nm, detection 530–630 nm) and photoreceptors were identified by Rh1-GFP expression (excitation 488 nm, detection 500–570 nm) as described [24, 47, 53]. Ommatidia lacking one or several RPCs lose their lozenge shape and are counted as affected ommatidia. Photoreceptor rhabdomeres were quantified using Fiji cell counter tool.

### Transmission electron microscopy of Drosophila eyes

Dissected *Drosophila* eyes were fixed in 0.1 M cacodylate buffer, 2.5% glutaraldehyde, and 2 mM CaCl_2_ for 16 h at 4°C. After rinsing with 0.1 M cacodylate buffer at RT, the eyes were incubated with 1% OsO_4_ in 0.1 M cacodylate buffer for 2 h at RT. Tissues were then progressively dehydrated in acetone at RT and mounted in 100% epoxy resin (Epon 812) in silicone-embedding molds. After resin polymerization for 48 h at 60°C, samples were sliced into 60 nm sections, which were stained with lead citrate and examined with a Philips CM120 transmission electron microscope (TEM) operating at 80 kV. For quantification, only LDs were considered that were clearly localized within either dRPC or photoreceptors. Vesicles surrounded by double membrane layers were excluded.

### Mice

Transgenic mice overexpressing human *FATP1* (*hFATP1TG*) specifically in the RPCs (driven by the RPC-specific VDM2 promoter) were generated on a C57BL/6J genetic background as previously described [31]. The *hFATP1TG* mice were maintained on a standard 12 h light (90 lux)/12 h dark cycle at ~22°C and were fed *ad libitum* with a standard rodent diet. Mice were housed in facilities accredited by the French Ministry of Agriculture and Forestry (B‐34 172 36—March 11, 2010). Mice were euthanized by cervical dislocation and the eyes were enucleated and dissected.

### Co-staining of lipids, perilipin, and ATP synthase

Nile Red staining of lipids [54] was used alone or in conjunction with fluorescent protein immunostaining. The eyes of 3-month-old mice were enucleated and the retina was separated from the RPC/choroid layer to obtain an empty eyeball [31]. The eyes were fixed in 4% PFA for 1 h at RT, washed with PBS, and permeabilized with 0.1% sodium dodecyl sulfate. They were then incubated for 20 min at RT in blocking buffer (10% fetal calf serum in PBS), and incubated overnight at 4°C with a mouse anti-mouse ATP synthase (Millipore MAB3494, 1:500) or rabbit anti-mouse perilipin (D1D8, Cell Signaling Technology # 9349, 1: 200) primary antibody. The tissues were then incubated for 4 h at RT with Alexa Fluor 488-conjugated anti-rabbit or anti-mouse secondary antibodies diluted in blocking buffer. The immunostained eyeballs were gently rinsed in PBS, incubated with Nile Red solution (10 μg/ml) for 30 min at RT in the dark, washed twice in PBS for 5 min at RT, and incubated with 4′,6-diamidino-2-phenylindole (DAPI, 1:1000) for 5 min at RT. The eyeballs were finally rinsed 5 times in PBS for 5 min at RT and mounted in Dako mounting medium. Confocal imaging was performed with a Zeiss LSM 5 LIVE DUO Highspeed/Spectral Confocal system. Images were acquired with Zeiss Zen software, and LDs were counted with ImageJ software.

### Quantification of neutral lipids in mouse retina

Mice were euthanized by vertebral dislocation, the eyes were enucleated, and the neural retina was removed from the eye cup. Neural retinas were directly frozen in liquid nitrogen. The RPC/choroid was scraped and collected in PBS, and the samples were centrifuged to remove the PBS. Tissues were stored dry at −80°C.

Lipids were extracted and analyzed as previously described [55]. Total lipids were extracted twice from tissues with ethanol/chloroform (1:2, v/v). Before extraction, internal standards were added. The organic phases were dried under nitrogen and lipid classes were separated by thin-layer chromatography on silica gel G using a mixture of hexane-ethyl ether -acetic acid (80:20:1 v/v/v). Lipids were transmethylated and the fatty acid methylesters were analyzed by gas chromatography. Briefly, samples were treated with toluene-methanol (1:1, v/v) and boron trifluoride in methanol. Transmethylation was carried out at 100°C for 90 min in screw-capped tubes. After addition of 1.5 mL, 10% K_2_CO_3_ in water, the resulting fatty acid methyl esters were extracted with 2 mL of isooctane and analyzed by gas chromatography, using an HP6890 instrument equipped with a fused silica capillary BPX70 SGE column (60 × 0.25 mm). The vector gas was hydrogen. The temperatures of the Ross injector and the flame ionization detector were set at 230°C and 250°C, respectively.

### Oxygen consumption

Respiration was measured on RPC/choroid and neural retinas permeabilized by incubation for 2 min with 15 μg digitonin per mg and resuspended in a respiratory buffer (pH 7.4, 10 mM KH_2_PO_4_, 300 mM mannitol, 10 mM KCl and 5 mM MgCl_2_). The respiratory rates were recorded at 37°C in 2 ml glass chambers using a high-resolution Oxygraph respirometer (Oroboros, Innsbruck, Austria). Assays were initiated in the presence of 5 mM malate/0.2 mM octanoyl carnitine to measure state 2, basal respiration (EIImo basal). Complex I-coupled state 3 respiration was measured by adding 0.5 mM NAD^+^/1.5 mM ADP (EIIImo β-ox). Then, 5 mM pyruvate and 10 mM succinate were added to reach maximal coupled respiration (EIIImpgso CxI+CxII), and 10 μM rotenone was injected to obtain the CII-coupled state 3 respiration. Oligomycin (8 μg/mL) was added to determine the uncoupled state 4 respiration rate. Finally, carbonyl cyanide-4-(trifluoromethoxy) phenylhydrazone (1 μM) was added to control the permeabilization of the tissues.

### Mitochondrial enzymatic activities

The activities of the mitochondrial citrate synthase (CS), oxoglutarate dehydrogenase (OGDH), isocitrate dehydrogenase (IDH) and fumarase were measured in 5 μl RPC/choroid and neural retina homogenates (sonicated in 50 μl PBS) at 37°C using a Beckman DU-640B spectrophotometer (Beckman Coulter) or a CLARIOstar (BMG LabTech) [56]. Briefly, citrate synthase activity was measured using 0.15 mM DTNB reagent (SIGMA Aldrich) which interacts with CoA-SH to produce TNB. The formation of TNB was followed for 1.5 min at wavelength of 412 nm. IDH and OGDH activities were determined in the presence of respective substrates isocitrate and α-ketoglutarate, by monitoring for 3 min the change in NAD^+^ to NADH which absorbs light at 340 nm. Fumarase activity was determined by measuring the conversion of L-malate to fumarate, monitoring the increase in absorbance at 250 nm. The optical density variation per minute is calculated from the curve and the enzymatic activity expressed as nmol of product formed/minute/mg protein.

### Electron microscopy of mouse RPCs

The eyes of 3–6 month-old *hFATP1TG* mice were rapidly enucleated, the corneas were removed, and the eyeballs were fixed by immersion in 2.5% glutaraldehyde in Sorensen’s phosphate buffer (0.1 M, pH 7.4) overnight at 4°C. The tissues were then rinsed in Sorensen’s buffer and post-fixed in 1% OsO_4_ for 2 h in the dark at RT. The tissues were rinsed twice, dehydrated in a graded series of ethanol solutions (30–100%), and embedded in EmBed 812 using a Leica EM AMW (Automated Microwave Tissue Processor for Electronic Microscopy). Sections (60 nm thick) were cut near the optic nerve (Leica-Reichert Ultracut E), counterstained with uranyl acetate, and examined using a Hitachi 7100 TEM (Centre de Ressources en Imagerie Cellulaire de Montpellier, France). The thickness of Bruch’s membrane was determined by measuring both the thickest and the thinnest parts of 5 fields throughout the retinal section per mouse. Data are presented as the median value per eye. We also enumerated the vacuoles in 5 fields of retinal sections per mouse at 10,000× magnification.

## Supporting information

S1 Fig Schematic of the mouse and *Drosophila* retina. (**A**) Mouse retinal pigment epithelial cells (mRPCs, pink) support photoreceptor rods and cones (green) by providing them with nutrients, transported across Bruch’s membrane from the underlying vasculature. (**B**) Tangential (top) and horizontal (bottom) views through a *Drosophila* ommatidium (of ~800 in total) showing *Drosophila* retinal pigment cells (dRPCs, primary, secondary and tertiary pigment in pink), bristle cells (cone cells are not represented on these drawings) organization around the photoreceptors. In contrast, to mRPCs that are only in contact with photoreceptor outer segments containing disk-filled of opsins (equivalent to rhabdomeres in flies), dRPCs are in contact with the cell bodies of photoreceptors and have a large zone of exchange.
(TIF)

S2 Fig *dFatp* overexpression with the *54C-Gal4* driver induces a specific accumulation of LDs in dRPCs. **(A-A” and B-B”)** Expression pattern of *54C-Gal4* driver. Expression of the *UAS-GFP* is driven by the *54C-Gal4*, GFP is visualized by confocal fluorescence microscopy on a pupal eye disc at 42 h after puparium formation (AFP). (A) merge, (A’) anti-GFP (A”) and anti-Elav antibody stainings. (A-A”) A strong expression of the GFP in secondary and tertiary pigment cells (dRPCs, arrows), and a weak expression in photoreceptors (Elav positive, arrowheads) at the level of Elav positive nuclei in the apical part of the pupal retina. (B-B”) A strong expression of GFP in six secondary (false color in brown) and three tertiary (false color in blue) pigment cells per ommatidium at the basal part of the eye disc. The position of bristle cells that lack GFP expression is shown (*) in A and B. GFP staining in primary pigment cells located above the photoreceptor layer is not apparent in these optical sections. (**C**) LD labeled with BODIPY^493/503^ (D3922) are revealed by confocal microscopy in horizontal sections of whole retinas from one-day-old flies expressing (a, c) *UAS-LacZ* (control) and (b, d) *UAS-dFatp* under the control of *54C-Gal4* driver alone (a, b) or concomitantly with *repo-Gal80* (c, d). Photoreceptors are counterstained with phalloidin-rhodamine (red). (**D**) Quantification of BODIPY^493/503^ (D3922) from the images shown in (C). Data are presented as the fold change in fluorescence intensity (dots/μm^2^) compared with the LacZ-control flies. Log adjusted values: *p<0.05, **p<0.01, ***p<0.001 by Tukey’s HSD paired sample comparison test.
(TIF)

S3 Fig Vitamin A is not required for *dFatp*-dependent accumulation of LD. (**A**) Quantification of the substantial loss of photoreceptors in whole-eye *dFatp*^*k10307*^ clone, quantified as % affected rhabdomeres. Whole-eye *dFatp*^*k10307*^ mutant flies were generated using the GMR-hid/FLP-FRT technique [49] with the following genotype *FRT40A- P{LacW}dFatpk*^*10307*^*/FRT40A-GMR-hid; ey-Gal4*, *UAS-FLP/TM6B*. For control flies a FRT40A-wild type chromosome was used. Control and *dFatp* mutant flies were fed on a Vitamin A deficient diet, which rescued photoreceptor degeneration in *dFatp* mutant. (**B**) LDs labeled with BODIPY^493/503^ (green) were revealed by confocal microscopy in horizontal sections of whole retinas from one-day-old flies expressing *UAS-dFatp* (b) or *UAS-LacZ* (a) under the control of *54C-Gal4* driver in flies fed with Vitamin A deficient diet. Photoreceptors are counterstained with phalloidin-rhodamine (red). Under vitamin A deficient diet (a, b), accumulation of LDs still occurs. (**C**) Quantification of BODIPY^493/503^ from the images shown in (B). Data are presented as the fold change in fluorescence intensity (dots/μm^2^) compared with the *LacZ*-control flies. Log adjusted values: **p<0.01, by Tukey’s HSD paired sample comparison test. (**D**) The *dFatp*^*k10307*^ loss of function allele eliminates LD content in the *Drosophila* retina of flies fed with a regular diet. Horizontal sections of wild-type (Control) or *dFatp*^*k10307*^ mutant retinas, stained with BODIPY^500/510^-C_12_ (green) to visualize LDs. Retinas of one-day-old flies were used, photoreceptors rhabdomeres were stained with phalloidin-rhodamine (red) and images were acquired by confocal fluorescence microscopy (scale bars 25 μm). Whole-eye *dFatp*^*k10307*^ mutant flies were generated using the GMR-hid/FLP-FRT technique with the following genotype *FRT40A-P{LacW}dFatp*^*k10307*^*/FRT40A-GMR-hid; ey-Gal4*, *UAS-FLP/TM6B*. For control flies a FRT40A-wild type chromosome was used.
(TIF)

S4 Fig TEM of retina from flies overexpressing the Bmm lipase. TEM showing ommatidia from one-day-old flies expressing *UAS-Brummer* under control of the dRPC-specific (*54C-Gal4*) driver. One ommatidium in each panel shows seven photoreceptors (false colored green) with central rhabdomeres surrounded by dRPCs (false colored pink). Scale bars, 2 μm (a–d), 1 μm (c’, d’). m, mitochondria; R rhabdomeres; arrowhead, small round shaped vesicles with an irregular shape and clear content that are distinct from LD.
(TIF)

S5 Fig *dFatp* is a functional ortholog of human *FATP1*. (**A, B**) Confocal fluorescence microscopy of retinas from *dFatp*^-/-^ (*dFatp*^*k10307*^) mutant flies without (A) or with (B) photoreceptor-specific expression of human *FATP1* (*dFatp*^-/-^;*Rh1>hFATP1*). *hFATP1* rescues the loss of photoreceptors in *dFatp*^-/-^ mutant clones. Retinas were generated using the Tomato/GFP-FLP/FRT technique [47], in which all photoreceptors are marked by GFP, and homozygous mutant mosaic retina is marked by the absence of TdTomato. *dFatp*^*k10307*^-mutant tissue shows loss of photoreceptors at 15 days of age (**A**). Scale bars, 10 μm. (**C**) Quantification of affected rhabdomeres, as shown in (A) and (B). Mean ± SD of n = 12 retinas. ***p<0.001 by two-sample t-test.
(TIF)

S6 Fig Knock-down of *Mdy* but not *Bmm-lipase* leads to photoreceptor degeneration. (**A**) LD labeled with BODIPY^493/503^ (green) were revealed by confocal microscopy in horizontal optical section of whole mount retinas from one-day-old flies expressing (a) *UAS-LacZ* RNAi (control), (b) *UAS-Bmm-lipase-RNAi* or (c) *UAS-Mdy-RNAi* under the control of a pan-retinal *ey-Gal4/GMR-Gal4* driver. Photoreceptors were counterstained with phalloidin-rhodamine (red). (**B)** Quantification of BODIPY^493/503^ staining from the images shown in (A). Data are presented as the fold change in fluorescence intensity (dots/μm^2^) compared with the *LacZ RNAi*-control flies. Mean ± SD of n = 4–10 flies/condition, **p<0.01 by Tukey’s HSD paired sample comparison test. (**C**) Tangential images of retinas of 20-day-old flies expressing Rh1-GFP visualized by the cornea neutralization method, carrying (a) *UAS-LacZ* RNAi (control), (b) *UAS-Bmm-lipase*-RNAi or (c) *UAS-Mdy*-RNAi induced by *ey-Gal4/GMR-Gal4*. *Bmm-lipase* knock-down does not affect photoreceptor survival, as indicated by intact rhabdomeres (b), while Mdy knock-down induces the loss of rhabdomeres (arrows in c). Scale bars, 10 μm. (**D**) Quantification of ommatidia with missing photoreceptor (affected ommatidia), as shown in (C). Mean ± SD of n = 5–11 flies. ***p<0.001 by two-sample t-test.
(TIF)
