# Supplementary figures and images for: Physiological and pathological roles of FATP-mediated lipid droplets in Drosophila and mice retina
Source: PLoS Genet. 2018 Sep 10;14(9):e1007627. doi: 10.1371/journal.pgen.1007627 (PMC6147681; doi:10.1371/journal.pgen.1007627)

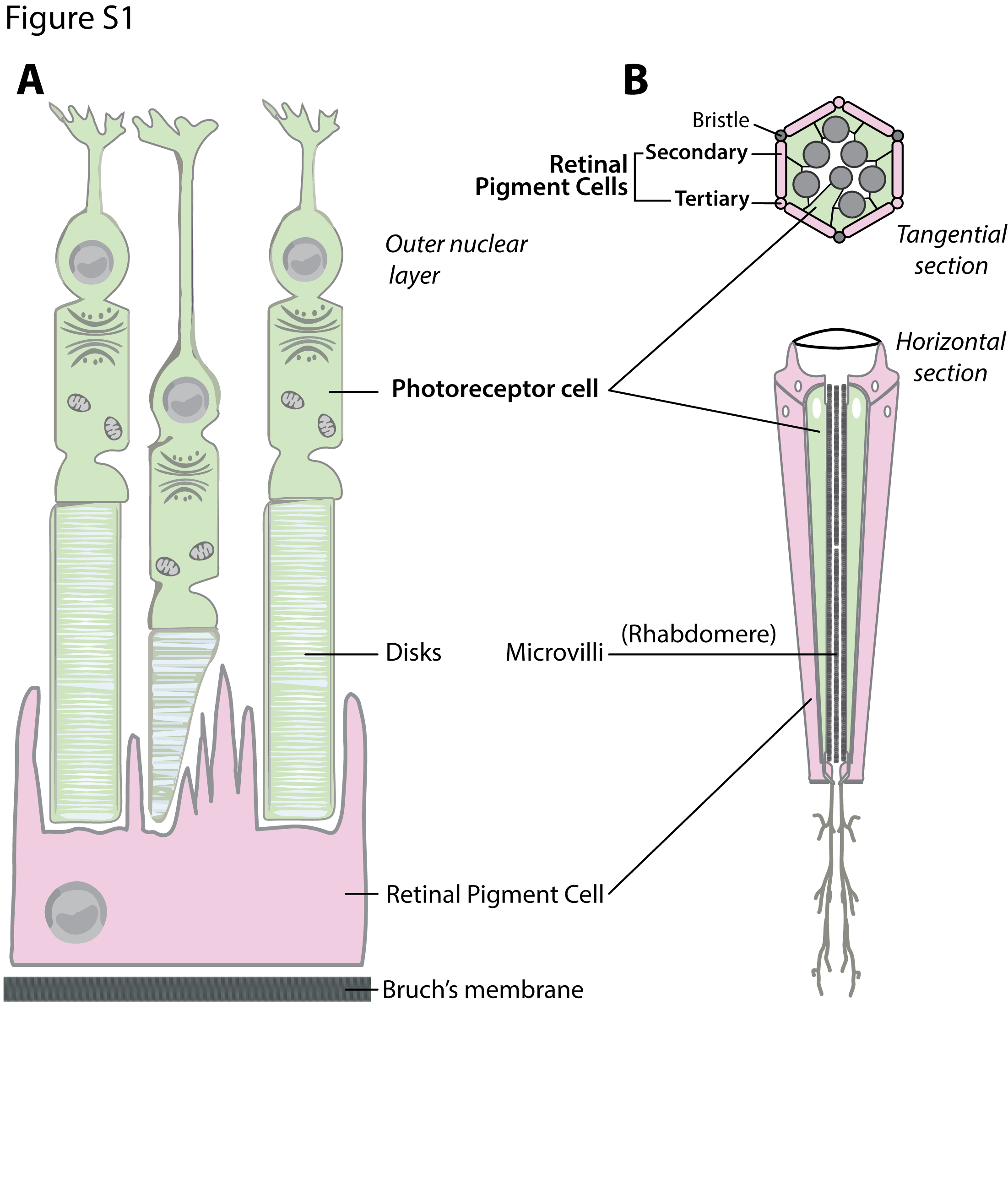

Supplement: S1 Fig — (A) Mouse retinal pigment epithelial cells (mRPCs, pink) support photoreceptor rods and cones (green) by providing them with nutrients, transported across Bruch’s membrane from the underlying vasculature. (B) Tangential (top) and horizontal (bottom) views through a Drosophila ommatidium (of ~800 in total) showing Drosophila retinal pigment cells (dRPCs, primary, secondary and tertiary pigment in pink), bristle cells (cone cells are not represented on these drawings) organization around the photoreceptors. In contrast, to mRPCs that are only in contact with photoreceptor outer segments containing disk-filled of opsins (equivalent to rhabdomeres in flies), dRPCs are in contact with the cell bodies of photoreceptors and have a large zone of exchange. (TIF) [file pgen.1007627.s001.tif]

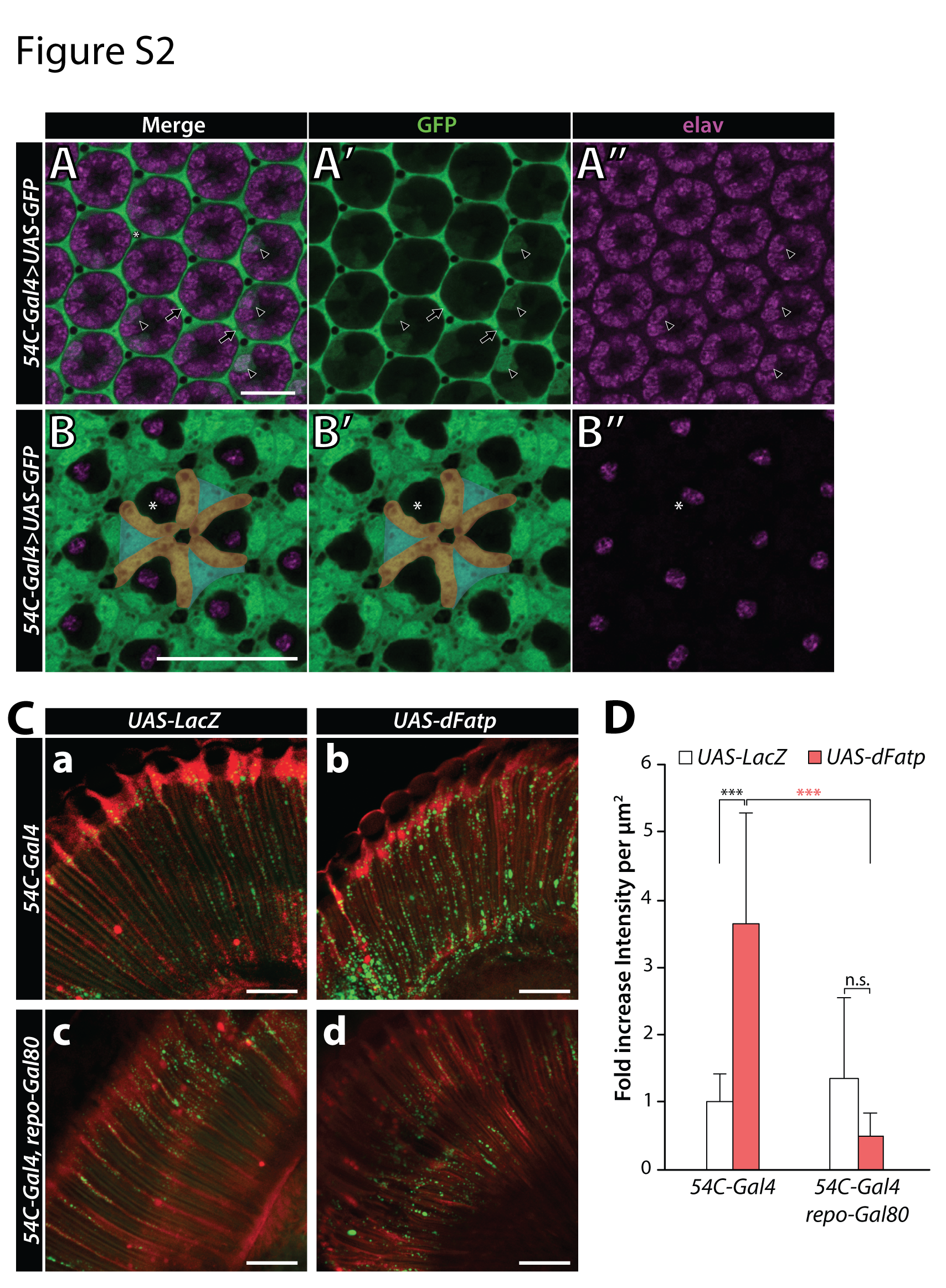

Supplement: S2 Fig — (A-A” and B-B”) Expression pattern of 54C-Gal4 driver. Expression of the UAS-GFP is driven by the 54C-Gal4, GFP is visualized by confocal fluorescence microscopy on a pupal eye disc at 42 h after puparium formation (AFP). (A) merge, (A’) anti-GFP (A”) and anti-Elav antibody stainings. (A-A”) A strong expression of the GFP in secondary and tertiary pigment cells (dRPCs, arrows), and a weak expression in photoreceptors (Elav positive, arrowheads) at the level of Elav positive nuclei in the apical part of the pupal retina. (B-B”) A strong expression of GFP in six secondary (false color in brown) and three tertiary (false color in blue) pigment cells per ommatidium at the basal part of the eye disc. The position of bristle cells that lack GFP expression is shown (*) in A and B. GFP staining in primary pigment cells located above the photoreceptor layer is not apparent in these optical sections. (C) LD labeled with BODIPY493/503 (D3922) are revealed by confocal microscopy in horizontal sections of whole retinas from one-day-old flies expressing (a, c) UAS-LacZ (control) and (b, d) UAS-dFatp under the control of 54C-Gal4 driver alone (a, b) or concomitantly with repo-Gal80 (c, d). Photoreceptors are counterstained with phalloidin-rhodamine (red). (D) Quantification of BODIPY493/503 (D3922) from the images shown in (C). Data are presented as the fold change in fluorescence intensity (dots/μm2) compared with the LacZ-control flies. Log adjusted values: *p<0.05, **p<0.01, ***p<0.001 by Tukey’s HSD paired sample comparison test. (TIF) [file pgen.1007627.s002.tif]

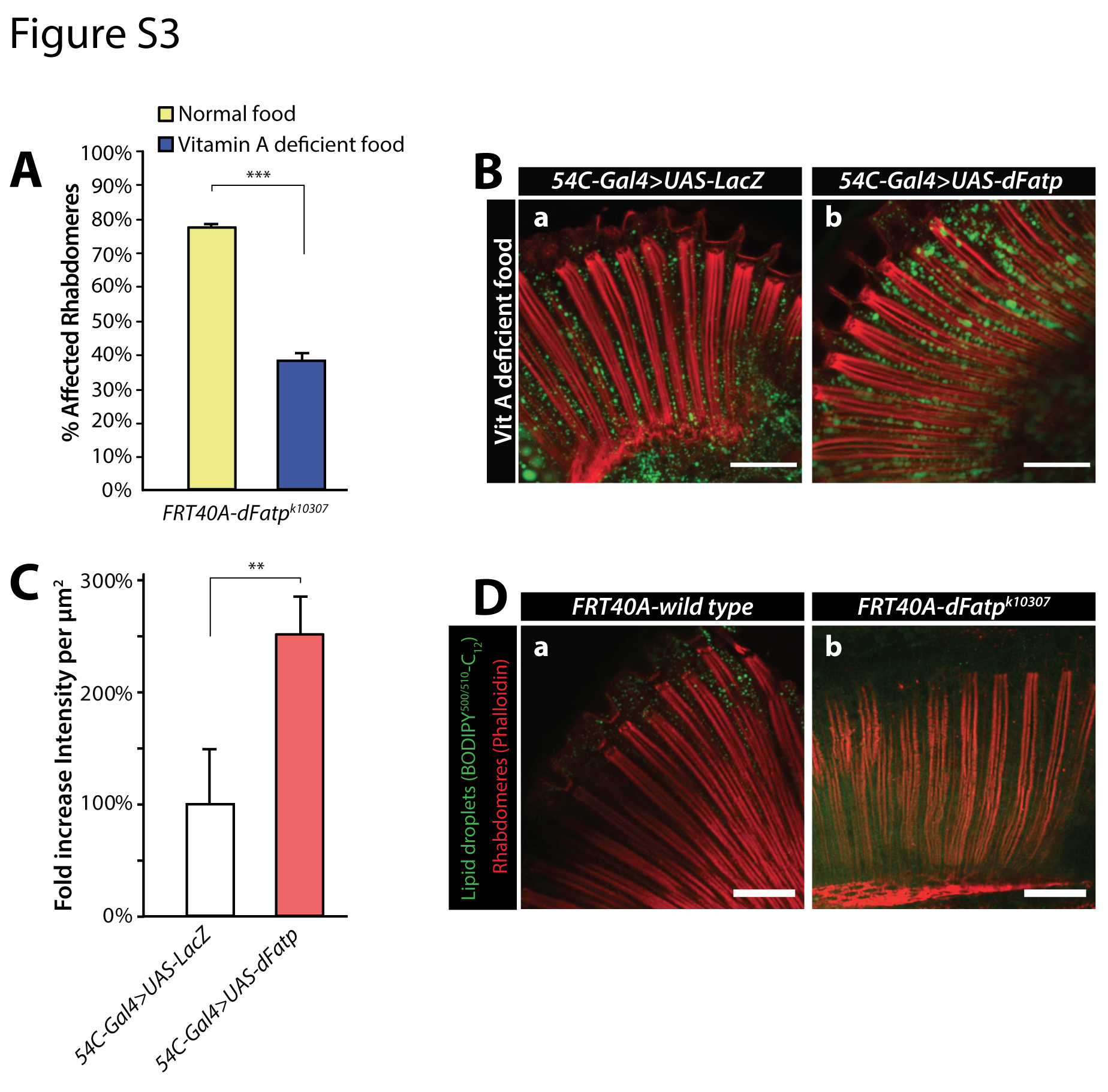

Supplement: S3 Fig — (A) Quantification of the substantial loss of photoreceptors in whole-eye dFatpk10307 clone, quantified as % affected rhabdomeres. Whole-eye dFatpk10307 mutant flies were generated using the GMR-hid/FLP-FRT technique [49] with the following genotype FRT40A- P{LacW}dFatpk10307/FRT40A-GMR-hid; ey-Gal4, UAS-FLP/TM6B. For control flies a FRT40A-wild type chromosome was used. Control and dFatp mutant flies were fed on a Vitamin A deficient diet, which rescued photoreceptor degeneration in dFatp mutant. (B) LDs labeled with BODIPY493/503 (green) were revealed by confocal microscopy in horizontal sections of whole retinas from one-day-old flies expressing UAS-dFatp (b) or UAS-LacZ (a) under the control of 54C-Gal4 driver in flies fed with Vitamin A deficient diet. Photoreceptors are counterstained with phalloidin-rhodamine (red). Under vitamin A deficient diet (a, b), accumulation of LDs still occurs. (C) Quantification of BODIPY493/503 from the images shown in (B). Data are presented as the fold change in fluorescence intensity (dots/μm2) compared with the LacZ-control flies. Log adjusted values: **p<0.01, by Tukey’s HSD paired sample comparison test. (D) The dFatpk10307 loss of function allele eliminates LD content in the Drosophila retina of flies fed with a regular diet. Horizontal sections of wild-type (Control) or dFatpk10307 mutant retinas, stained with BODIPY500/510-C12 (green) to visualize LDs. Retinas of one-day-old flies were used, photoreceptors rhabdomeres were stained with phalloidin-rhodamine (red) and images were acquired by confocal fluorescence microscopy (scale bars 25 μm). Whole-eye dFatpk10307 mutant flies were generated using the GMR-hid/FLP-FRT technique with the following genotype FRT40A-P{LacW}dFatpk10307/FRT40A-GMR-hid; ey-Gal4, UAS-FLP/TM6B. For control flies a FRT40A-wild type chromosome was used. (TIF) [file pgen.1007627.s003.tif]

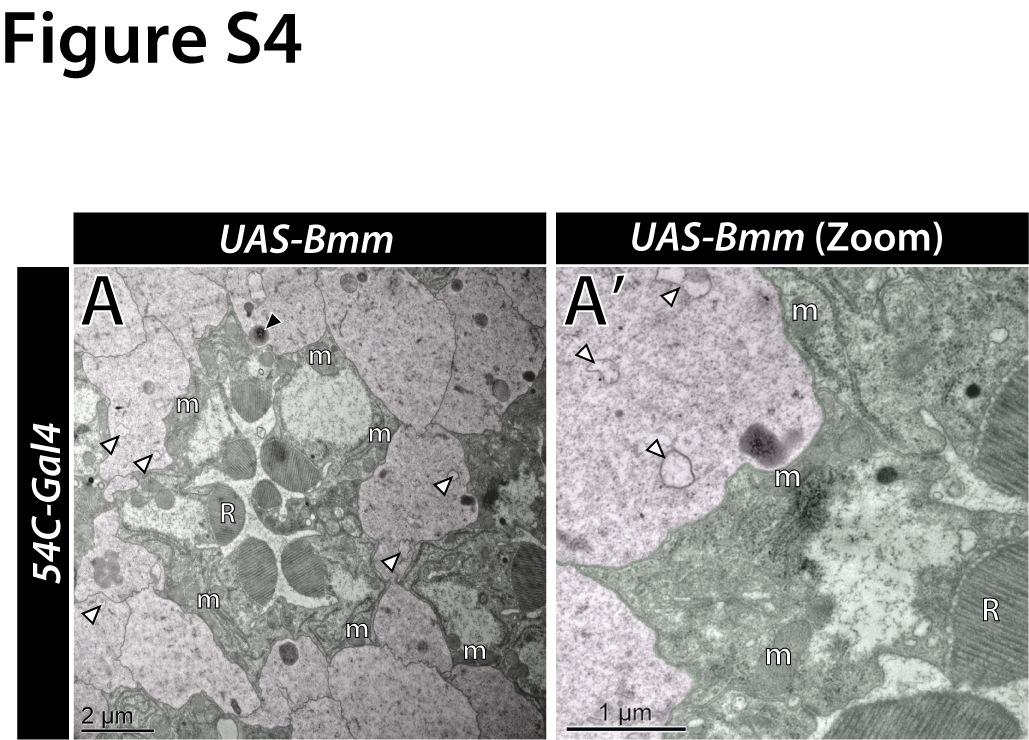

Supplement: S4 Fig — TEM showing ommatidia from one-day-old flies expressing UAS-Brummer under control of the dRPC-specific (54C-Gal4) driver. One ommatidium in each panel shows seven photoreceptors (false colored green) with central rhabdomeres surrounded by dRPCs (false colored pink). Scale bars, 2 μm (a–d), 1 μm (c’, d’). m, mitochondria; R rhabdomeres; arrowhead, small round shaped vesicles with an irregular shape and clear content that are distinct from LD. (TIF) [file pgen.1007627.s004.tif]

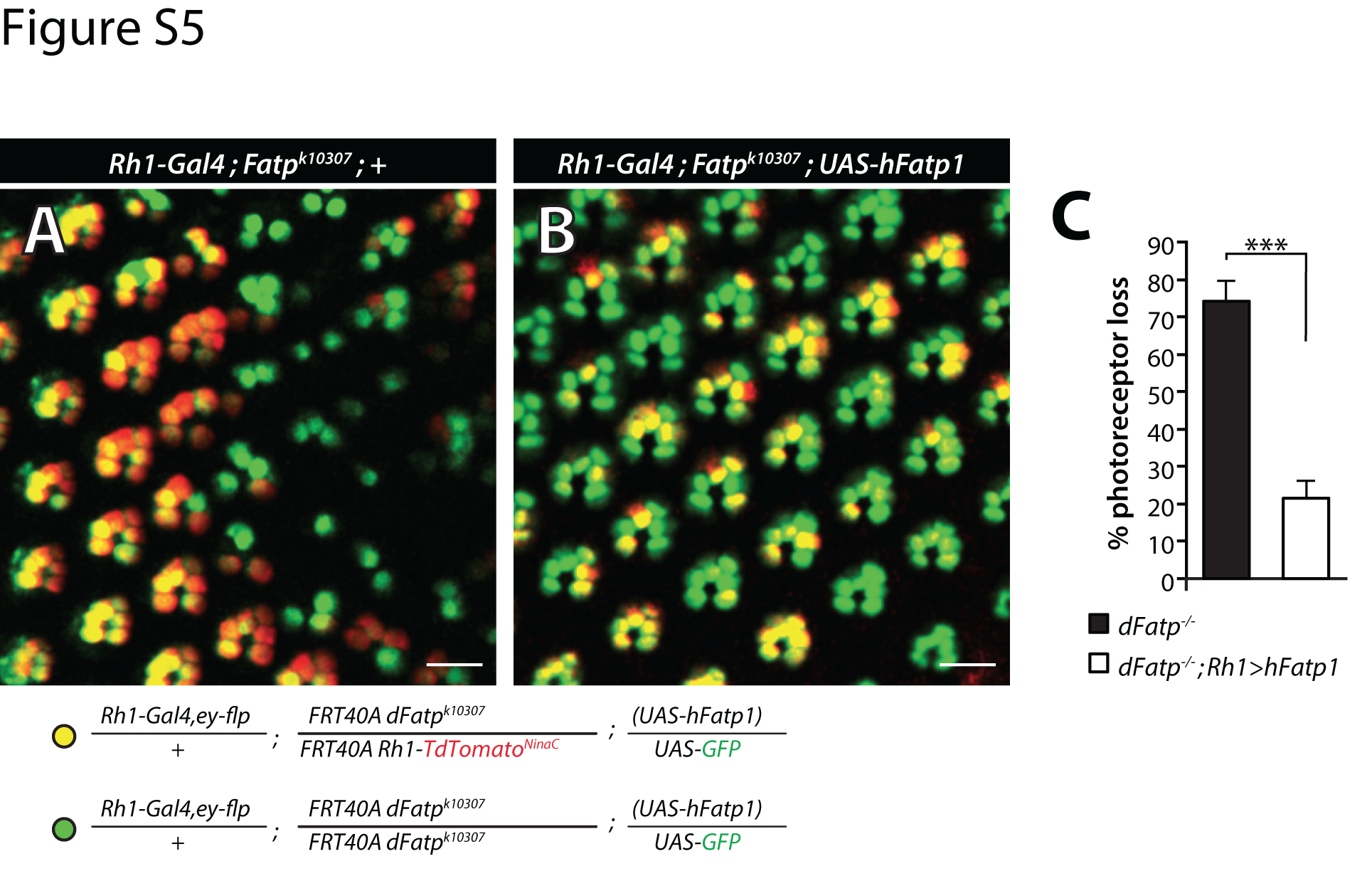

Supplement: S5 Fig — (A, B) Confocal fluorescence microscopy of retinas from dFatp-/- (dFatpk10307) mutant flies without (A) or with (B) photoreceptor-specific expression of human FATP1 (dFatp-/-;Rh1>hFATP1). hFATP1 rescues the loss of photoreceptors in dFatp-/- mutant clones. Retinas were generated using the Tomato/GFP-FLP/FRT technique [47], in which all photoreceptors are marked by GFP, and homozygous mutant mosaic retina is marked by the absence of TdTomato. dFatpk10307-mutant tissue shows loss of photoreceptors at 15 days of age (A). Scale bars, 10 μm. (C) Quantification of affected rhabdomeres, as shown in (A) and (B). Mean ± SD of n = 12 retinas. ***p<0.001 by two-sample t-test. (TIF) [file pgen.1007627.s005.tif]

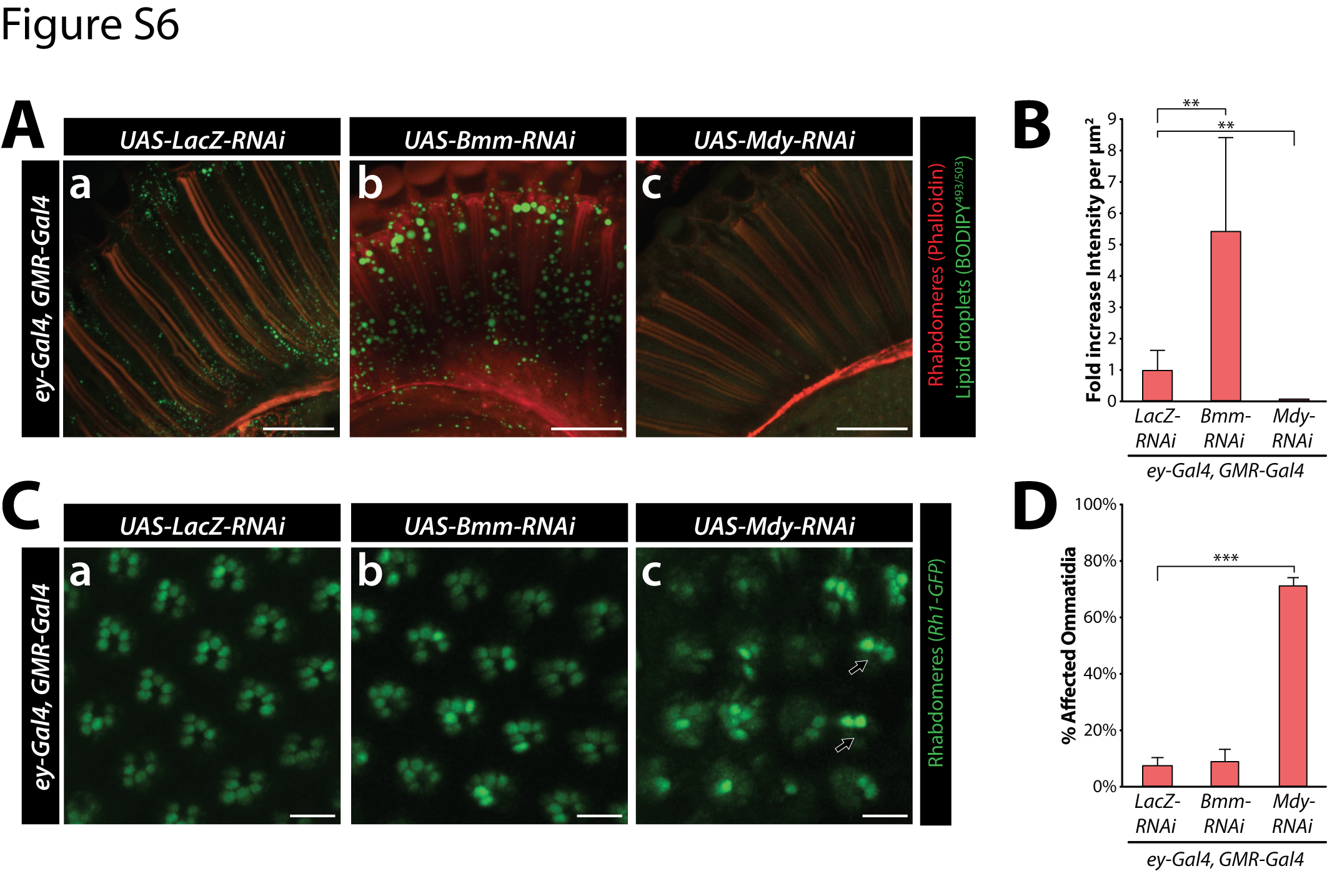

Supplement: S6 Fig — (A) LD labeled with BODIPY493/503 (green) were revealed by confocal microscopy in horizontal optical section of whole mount retinas from one-day-old flies expressing (a) UAS-LacZ RNAi (control), (b) UAS-Bmm-lipase-RNAi or (c) UAS-Mdy-RNAi under the control of a pan-retinal ey-Gal4/GMR-Gal4 driver. Photoreceptors were counterstained with phalloidin-rhodamine (red). (B) Quantification of BODIPY493/503 staining from the images shown in (A). Data are presented as the fold change in fluorescence intensity (dots/μm2) compared with the LacZ RNAi-control flies. Mean ± SD of n = 4–10 flies/condition, **p<0.01 by Tukey’s HSD paired sample comparison test. (C) Tangential images of retinas of 20-day-old flies expressing Rh1-GFP visualized by the cornea neutralization method, carrying (a) UAS-LacZ RNAi (control), (b) UAS-Bmm-lipase-RNAi or (c) UAS-Mdy-RNAi induced by ey-Gal4/GMR-Gal4. Bmm-lipase knock-down does not affect photoreceptor survival, as indicated by intact rhabdomeres (b), while Mdy knock-down induces the loss of rhabdomeres (arrows in c). Scale bars, 10 μm. (D) Quantification of ommatidia with missing photoreceptor (affected ommatidia), as shown in (C). Mean ± SD of n = 5–11 flies. ***p<0.001 by two-sample t-test. (TIF) [file pgen.1007627.s006.tif]
